# Insularity determines nestling sex ratio variation in Egyptian vulture populations

**DOI:** 10.1002/ece3.10371

**Published:** 2023-07-29

**Authors:** Guillermo Gómez‐López, Ana Sanz‐Aguilar, Martina Carrete, Eneko Arrondo, José Ramón Benítez, Olga Ceballos, Ainara Cortés‐Avizanda, Félix de Pablo, José Antonio Donázar, Óscar Frías, Laura Gangoso, Marina García‐Alfonso, José Luis González, Juan Manuel Grande, David Serrano, José Luis Tella, Guillermo Blanco

**Affiliations:** ^1^ Department of Evolutionary Ecology, National Museum of Natural Sciences Spanish National Research Council Madrid Spain; ^2^ Department of Biodiversity, Ecology and Evolution, Faculty of Biology Complutense University of Madrid Madrid Spain; ^3^ Animal Demography and Ecology Group Institut Mediterrani d'Estudis Avançats, Spanish National Research Council Mallorca Spain; ^4^ Applied Zoology and Conservation Group Universitat de les Illes Balears Palma Spain; ^5^ Department of Physical, Chemical and Natural Systems Pablo de Olavide University Sevilla Spain; ^6^ Department of Zoology University of Granada Granada Spain; ^7^ Department of Applied Biology Miguel Hernández University Elche Spain; ^8^ Department of Biodiversity Agencia de Medioambiente y Agua, Junta de Andalucía Sevilla Spain; ^9^ UGARRA Pamplona Spain; ^10^ Department of Plant Biology and Ecology University of Seville Sevilla Spain; ^11^ Department of Conservation Biology Doñana Biological Station, Spanish National Research Council Sevilla Spain; ^12^ Department of Environment and Biosphere Reserve Consell Insular de Menorca, Plaza Maó Spain; ^13^ ColBEC, INCITAP‐CONICET‐UNLPam/FCEyN‐UNLPam Santa Rosa Argentina

**Keywords:** demography, islands, *Neophron percnopterus*, nestling sex, offspring sex ratio, sex sequence

## Abstract

Variation in offspring sex ratio, particularly in birds, has been frequently studied over the last century, although seldom using long‐term monitoring data. In raptors, the cost of raising males and females is not equal, and several variables have been found to have significant effects on sex ratio, including food availability, parental age, and hatching order. Sex ratio differences between island populations and their mainland counterparts have been poorly documented, despite broad scientific literature on the island syndrome reporting substantial differences in population demography and ecology. Here, we assessed individual and environmental factors potentially affecting the secondary sex ratio of the long‐lived Egyptian vulture *Neophron percnopterus*. We used data collected from Spanish mainland and island populations over a ca. 30‐year period (1995–2021) to assess the effects of insularity, parental age, breeding phenology, brood size, hatching order, type of breeding unit (pairs vs. trios), and spatial and temporal variability on offspring sex ratio. No sex bias was found at the population level, but two opposite trends were observed between mainland and island populations consistent with the island syndrome. Offspring sex ratio was nonsignificantly female‐biased in mainland Spain (0.47, *n* = 1112) but significantly male‐biased in the Canary Islands (0.55, *n* = 499), where a male‐biased mortality among immatures could be compensating for offspring biases and maintaining a paired adult sex ratio. Temporal and spatial variation in food availability might also have some influence on sex ratio, although the difficulties in quantifying them preclude us from determining the magnitude of such influence. This study shows that insularity influences the offspring sex ratio of the Egyptian vulture through several processes that can affect island and mainland populations differentially. Our research contributes to improving our understanding of sex allocation theory by investigating whether sex ratio deviations from parity are possible as a response to changing environments comprised by multiple and complexly interrelated factors.

## Graphical abstract

We assessed individual and environmental factors potentially affecting the offspring sex ratio of the Egyptian vulture (*Neophron percnopterus*), collected from Spanish mainland and island populations over a ca. 30‐year period (1995–2021). Offspring sex ratio was nonsignificantly female‐biased in mainland Spain while significantly male‐biased in the Canary Islands, where a male‐biased mortality among immatures could be compensating for offspring biases and maintaining a paired adult sex ratio.

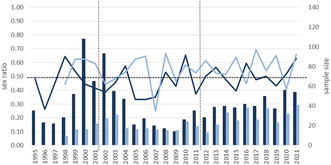

## 1 INTRODUCTION

Long‐term studies are essential for understanding the ecological and evolutionary processes that operate in wild populations (Sheldon et al., 2022). Variation in offspring sex ratio has been frequently addressed over the last century (Mayr, 1939; Payevsky, 2021), although rarely using long‐term monitoring data (Rosenfield et al., 2015; Warkentin et al., 2022). In birds, offspring sex ratio can be categorized as primary or secondary depending on whether it refers to the proportion of males over females at fertilization or at hatching, respectively (Mayr, 1939). Following Fisher's sex allocation theory (Fisher, 1930), numerous studies have confirmed that offspring sex ratio tends to be 1:1 in different species and populations (e.g., Clutton‐Brock, 1986; Donald, 2007; Ellegren et al., 1996; Gómez‐López et al., 2022; Gowaty, 1993). Although Fisher's statement of sex ratio parity is based on the idea that the cost of rearing male and female offspring is the same (Fisher, 1930), this is not true in species that exhibit sexual size dimorphism (Komdeur & Pen, 2002; Navara, 2018; Szász et al., 2012), which would cause differential parental investment in male and female offspring and, therefore, a biased sex ratio (Szász et al., 2012). Additionally, environmental conditions during breeding, such as weather or food availability, may add variation to the costs of producing offspring of different sexes and thus affect offspring sex ratio, favoring a greater investment in the sex that maximizes parental fitness (Trivers & Willard, 1973).

Facultative parental manipulation of the primary sex ratio (Alonso‐Alvarez, 2006; Pike & Petrie, 2003; West et al., 2002) and differential egg or chick mortality during the period between laying and fledging (Bradbury & Blakey, 1998; Nager et al., 2000; Székely et al., 2006) are the two main mechanisms that can bias the offspring sex ratio. Despite intensive research over the last decades, the processes underlying parental manipulation or differential early mortality remain poorly understood (Navara, 2018) and the range of environmental and individual factors influencing both mechanisms has proven to be broad, complex, and interconnected (Hasselquist & Kempenaers, 2002; West et al., 2002).

Raptors, specifically, are characterized by a reversed sexual dimorphism, with females being the larger sex and thus tending to have higher growth requirements and parental investment (Anderson et al., 1993; Frumkin, 1989; Riedstra et al., 1998). Offspring sex ratio in raptors is often biased by food availability, with poor years often associated with higher production of males, the less costly sex (Arroyo, 2002; Dzus et al., 1996; Wiebe & Bortolotti, 1992). The age of breeders, which is closely related to their experience and performance (Curio, 1983; Forslund & Pärt, 1995), also seems to influence offspring sex ratio in raptors, with younger and less experienced breeders tending to raise males more often (Blank & Nolan, 1983; Ferrer et al., 2009; Warkentin et al., 2022). Breeding timing can also affect offspring sex ratio, either by increasing the proportion of females at the beginning of the breeding season and that of males at the end (Mora et al., 2010; Ristow & Wink, 2004; Tschumi et al., 2019), or vice versa (Daan et al., 1996; Smallwood & Smallwood, 1998; Tella et al., 1996), depending on the species. Postnatal dispersal patterns may also cause biases in offspring sex ratio, with overproduction of the dispersing sex—females in birds, including raptors—being relatively common (Gowaty, 1993; Greenwood, 1980), especially under conditions of high conspecific density (Ferrer et al., 2009; Morandini et al., 2019). Moreover, the type of breeding unit (e.g., pairs or trios) can affect breeding success and population productivity (Carrete, Donázar, Margalida, & Bertran, 2006) and thus influence offspring sex ratio (Nisbet & Hatch, 1999). For instance, Bearded Vulture (*Gypaetus barbatus*) trios show a lower productivity than pairs, suggesting that the third breeding individual is costly (Carrete, Donázar, Margalida, & Bertran, 2006), even though there should be additional parental care provided by the subordinate (e.g., food provisioning; Bertran & Margalida, 2002).

Effects of brood size on offspring sex ratio have been documented in several raptor species, with greater biases in the smallest or largest broods, while intermediate‐sized broods tend to show a balanced sex ratio (Dijkstra et al., 1998; Warkentin et al., 2022). Most raptors also show asynchronous hatching, in which the oldest and thus largest nestling receives more food, irrespective of its sex (Slagsvold, 1990). Although this does not apparently favor a particular sex (Slagsvold et al., 1986), nestling sex has been found to vary according to the hatching order. In mixed‐sex broods, first‐hatched nestlings are usually females in some species, such as the Peregrine Falcon *Falco peregrinus* (Olsen & Cockburn, 1991), Montagu's Harrier *Circus pygargus* (Leroux & Bretagnolle, 1996), Eurasian Kestrel *Falco tinnunculus* (Blanco et al., 2003), Eleonora's Falcon *Falco eleonorae* (Xirouchakis et al., 2022), and Bald Eagle *Haliaeetus leucocephalus* (Bortolotti, 1986), and more often males in others such as the Harris' Hawk *Parabuteo unicinctus* (Bednarz & Hayden, 1991) and Scops Owl *Otus scops* (Blanco et al., 2002). These variations in first‐hatched nestlings are mainly due to parental control of the sex within the egg sequence and/or by food monopolization and siblicide by the first‐hatched nestling over the youngest (Bortolotti, 1986; Simmons, 1988). If the first nestling belongs to the larger sex, the second faces a double disadvantage—being smaller because of both sex and hatching order—although this can be compensated for by lower resource requirements or faster growth (Clutton‐Brock, 1986; Legge et al., 2001). Conversely, if the smaller sex hatches first, competitive interactions between siblings are likely to increase as intrabrood hierarchies are reversed (Legge et al., 2001). Rearing male‐only broods alternating with female‐only broods could be a way to avoid dominance problems derived from sexual size dimorphism and even hatching order (Bortolotti, 1986). Different combinations of sexes have been found in females producing broods of more than two nestlings, in some cases taken to the extreme (e.g., long unbroken sequences of the same sex over consecutive broods and years; Heinsohn et al., 1997).

Spatial differences in offspring sex ratio within species have also been addressed in the scientific literature, with some emphasis on the so‐called “island syndrome.” This phenomenon illustrates the unique traits of populations living on islands compared with mainland populations, such as morphology (e.g., size or shape), physiology (e.g., immune system), demography (e.g., fecundity, growth, survival, dispersal, or density), behavior (e.g., territoriality or aggressiveness), and general ecology (e.g., habitat niche, competition, or life‐history strategies; Blondel, 2000; Covas, 2016; Losos & Ricklefs, 2009; Whittaker & Fernández‐Palacios, 2007). In long‐lived land birds with deferred sexual maturity that occur both on islands and the mainland, this syndrome may lead to strong differences in their population dynamics. For example, many mainland migratory raptors become sedentary on islands (Donázar et al., 2005; Ferrer et al., 2011). In the absence of costs associated with migration or new threats at wintering areas, survival on islands can be higher than in mainland populations, especially when human‐related mortality factors are reduced (Badia‐Boher et al., 2019; Buechley et al., 2021; Sanz‐Aguilar et al., 2012; Sanz‐Aguilar, De Pablo, & Donázar, 2015; Sergio et al., 2014). Young individuals can also gain experience more quickly and breed for the first time earlier than their migratory counterparts, facilitating the persistence of island populations (Ferrer et al., 2004, 2011). On the contrary, populations inhabiting islands are usually small and thus particularly prone to inbreeding depression, which might result in lower genetic variability and greater susceptibility to environmental changes (Agudo et al., 2012; Kretzmann et al., 2003; Lande, 1988). The proportion of nonbreeding individuals is also generally higher in island than in mainland populations (Blanco et al., 2009; Donázar et al., 2002), and negative density‐dependent effects on survival and reproduction are also more likely in the former (Brouwer et al., 2009). All of these factors can affect offspring sex ratio in island populations. For instance, high conspecific densities, constrained by an absent or limited dispersal in islands, may cause more difficulties in raising offspring of the more costly sex since high‐quality territories might be scarcer (Bonal & Aparicio, 2008; Ferrer & Donazar, 1996). Venables and Brooke (2015), using an interspecific approach, showed male‐biased sex ratios in adult individuals of island versus mainland species. However, factors operating on adult sex ratio (e.g., mortality factors or dispersal ability) are not expected to be the same as those affecting offspring sex ratio. To our knowledge though, there are no studies addressing variation in offspring sex ratio in long‐lived avian species inhabiting both island and continental areas.

Here, we explored factors potentially affecting the secondary sex ratio in mainland and island breeding populations of the Egyptian vulture *Neophron percnopterus*, a long‐lived raptor that rears one or two nestlings per season. We assessed offspring sex ratio variation across regions, as well as the effects of breeding phenology, brood size, hatching order, parental age, type of breeding unit (pairs vs. trios), and food availability on the probability of a nestling being a male (see Table 1). Given that male Egyptian vultures are smaller and, presumably, the least costly sex to produce, we predict that more males would be produced (i) in island populations, (ii) in years with lower food availability, (iii) in breeding units formed by younger parents, (iv) as second‐hatched nestlings (in multiple broods), and (v) when hatching later in the breeding season. Contrary to most studies on variation in offspring sex ratio, we take advantage of long‐term monitoring programs (i.e., over the last 30 years) performed in mainland Spain, the Canary Islands and the Balearic Islands. Long‐term data allow us to determine sex ratio patterns that are influenced by seasonal and interannual changes like food availability, weather conditions and population density (Griggio et al., 2002), something that is not feasible from short‐term monitoring. Furthermore, short‐term studies are generally conducted with small sample sizes, which may yield nonsignificant results due to low statistical power and Type II errors (Rosenfield et al., 1996). Analyses of short‐term datasets may also involve Type I errors, since initially significant results may become nonsignificant once the sample increases substantially and interyear variability is considered (Hasselquist & Kempenaers, 2002). Hence, the effects of environmental and social factors that are operating in the long‐term, especially in long‐lived species, can only be detected with data collected over large temporal windows that allow us to understand the real importance of selective pressures operating on sex ratio adjustment (Rosenfield et al., 2015).

**TABLE 1:** Explanatory variables included on each scale of analysis and their description.

Variable name	Description	All^a^	Peninsular Spain^b^	Canary Islands^c^	Balearic Islands
Region	Breeding nucleus where nestlings were born (peninsular Spain: Andalusia, Aragon, Navarra, Segovia; islands: Canary Islands, Balearic Islands)	+	+		
Insularity	Mainland or island	+			
Territory	Territory where nestlings hatched	+	+	+	+
Year	Year of birth of nestlings	+	+	+	+
Mad‐cow crisis	Hatching period of nestlings (before, during or after the mad‐cow crisis)	+	+	+	+
Hatching date	Hatching date of nestlings (in Julian date)	+	+	+	
Brood size	Number of known nestlings comprising each brood (one or two)	+	+	+	+
Hatching order	Order of hatching of nestlings in each brood (single nestling, first‐hatched in double brood or second‐hatched in double brood)	+	+	+	
Breeding unit	Pair or trio			+	
Parental age	Age of parents (only banded breeders) at the year of birth of their nestling		+	+	
Conspecific density	Annual number of breeding pairs			+	+
Population trend	Difference between the number of breeding pairs in each year and the number of breeding pairs one to seven years earlier			+	

^a^ We excluded the Balearic Islands from the general analysis since no data on hatching date and order were available.

^b^ We performed an additional analysis for peninsular Spain using a data subset with the age of male and female breeders available only.

^c^ We performed an additional analysis for the Canary Islands using a data subset with the age of male and female breeders available only.

## 2 MATERIALS AND METHODS

### 2.1 Species and study area

The Egyptian vulture is an obligate avian scavenger that occupies open and rugged areas of the Circum‐Mediterranean, the Middle East, sub‐Saharan Africa, central Asia and India, as well as islands of the Indian Ocean, the Mediterranean Sea, and Macaronesia (Birdlife International, 2021). Its broad diet includes not only carcasses of domestic livestock and wild vertebrates but also organic waste, insects, eggs, and feces (Cramp & Simmons, 1980; Negro et al., 2002). Although it is a territorial species during breeding, individuals can congregate at feeding and roosting sites (Ceballos & Donázar, 1990). The European population is mainly migratory and overwinters in Africa, while the island populations are sedentary (Cramp & Simmons, 1980; Sanz‐Aguilar, De Pablo, & Donázar, 2015). Adults are highly philopatric, especially males (Grande, 2006; Serrano et al., 2021). It is a relatively small vulture (ca. 2 kg), and females are slightly larger and weigh 10%–15% more than males (Sanz‐Aguilar et al., 2017). Individuals typically nest on cliffs and form monogamous pairs, although polyandrous and polygynous trios can also occur (Tella, 1993; Van Overveld et al., 2020). Females lay one or two eggs between April and May. Incubation lasts ca. 42 days (Donázar et al., 1994), and nestlings become independent approximately 3 months after hatching (Donázar & Ceballos, 1990). There are no apparent differences in juvenile survival rates between sexes (Grande et al., 2009), but breeding females and young breeding males display lower survival compared with older breeding males in some populations (Sanz‐Aguilar et al., 2017). Despite some behavioral differences, both sexes invest similar parental effort throughout the breeding period (Donázar, 1993; Morant et al., 2019). Individuals from the Canary Islands also show a sexual asymmetry in foraging behavior that could be associated with intersex competition for resources, with females using supplementary feeding stations preferentially and males visiting more farms (Van Overveld et al., 2018). The species is listed as “Endangered” globally (Birdlife International, 2021) and as “Vulnerable” in Europe (Birdlife International, 2020), while the subspecies *N.p. majorensis*, endemic to the Canary Islands, is also considered “Endangered” (Spanish Royal Decree 139/2011).

### 2.2 Fieldwork and sampling procedures

**FIGURE 1:**
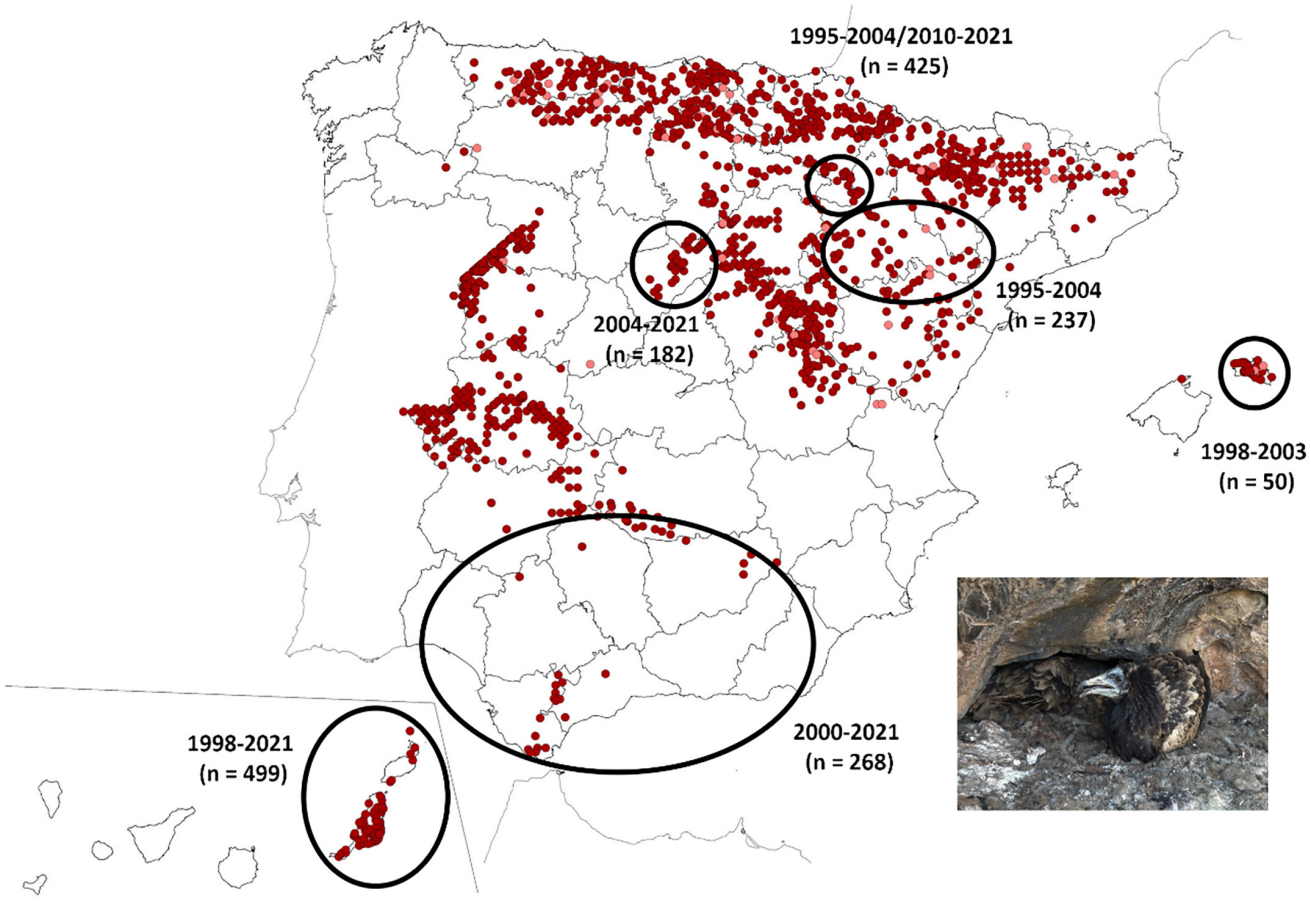
Location of the Egyptian vulture breeding regions (black circles) used for the present study in peninsular Spain and the Canary (Fuerteventura and Lanzarote) and Balearic (Menorca) Islands. Next to each region, note the study period and sample size. Dark red spots correspond to confirmed breeding pairs in the 2018 national census, while light red spots indicate pairs whose reproduction is likely. Figure modified from Del Moral and Molina (2018). Photo credit: Guillermo Blanco.

The present study took advantage of the long‐term monitoring programs of Egyptian vultures in northern, central, and southern Spain (Communities of Aragon, Navarra, Castilla y Leon, and Andalusia), as well as in the Canary and Balearic Islands, thus including some of the most important breeding nuclei in Spain (Figure 1). Populations were monitored from 1995 to 2021, although each region was surveyed over different periods (see Section 3). During part of each breeding season (April–May), fieldwork was carried out to detect territorial individuals and their identity and assess the type of breeding unit (pair: male and female, or trio: two males and one female or two females and one male) and whether they raise nestlings and their number (breeding success and brood size, respectively), when possible. As it is difficult to determine whether a single nestling belongs to a single brood or is the remaining nestling of a brood of two after brood reduction, we assumed that brood size was one when only one nestling was found in the nest. Observations were made with telescopes at long distances to minimize disturbances. From May to August, we accessed successful nests when nestlings were typically 45–65 days old to mark them with metal and plastic bands with alphanumeric codes, which allow individual identification at a distance. Hatching order was assessed in double broods according to differences in nestling body size (mainly weight, and wing and tail length) and plumage development. A blood sample was obtained from the brachial vein of each nestling and preserved in absolute ethanol for molecular sexing. Molecular sexing was done following Griffiths et al. (1996) for samples collected between 1995 and 1997, and Fridolfsson and Ellegren (1999) for samples collected from 1998 onwards. In those cases where brood reduction took place, it happened prior to the moment of banding, so no biases are expected in this regard. As we did not know the sex of nestlings that died before banding, our analyses focused on the secondary sex ratio.

### 2.3 Statistical analyses

We used Monte Carlo simulations to evaluate whether the sex ratios of the studied regions were significantly biased while controlling for differences in sample sizes. Briefly, for each breeding region, we ran 1000 simulations by randomly picking a number of nestlings equal to its sample size from a theoretical set of 3000 nestlings with a balanced sex ratio (1:1) and calculating the resulting sex ratio. Significance tests were generated by counting the number of randomized cases that resulted in a value equal to or greater/lower than the observed sex ratio of the region (compiling all years sampled) and then dividing by 1000 (i.e., the total number of randomizations; Serrano et al., 2008).

We ran univariate models (generalized linear models; GLMs) to explore the potential effect of the year (fixed factor) on the probability of a nestling being a male using four datasets, namely all nestlings pooled together, and nestlings from peninsular Spain, Canary Islands, and Balearic Islands analyzed separately. We then used generalized linear mixed models (GLMMs) to assess the effects of hatching date, natal region (i.e., peninsular Spain—Andalusia, Aragon, Navarra, Segovia; Islands—Canary Islands, Balearic Islands), brood size (one or two nestlings), hatching order (single, first, or second nestlings), the type of breeding unit (pair or trio; only for the Canary Islands), and food availability (i.e., period of the mad‐cow crisis, see below; Table 1) on the probability of a nestling being a male (logit link function; binomial error distribution; see a similar approach in Gómez‐López et al., 2022). Although we first assessed the effects of our explanatory variables on the offspring sex ratio of Egyptian vultures across the entire studied distribution, ecological and genetic differences between island and mainland populations, as well as between both islands, support the need to also perform separate models for peninsular Spain, the Canary Islands, and the Balearic Islands (Table 1). Hatching date and order could not be calculated for nestlings from the Balearic Islands as no biometrical data were available, so analyses for these islands were performed separately using GLMMs with brood size and food availability as explanatory variables only (Table 1). We estimated nestling age (in days) using two methods. For individuals banded in Navarra, Aragon, Andalusia, and the Canary Islands, the age of single and first‐hatched nestlings of double broods was estimated from a linear regression relating the length of the seventh primary to age (Donázar & Ceballos, 1989). Second‐hatched nestlings of double broods were considered to hatch 5 days later than their older sibling (Donázar & Ceballos, 1989). For Segovia (Castilla y Leon), since no data on primary length were available, we estimated nestling age, regardless of their hatching order, using a regression of the weight of nestlings of known age from the nearby broods in Navarra and Aragon (Figure S3). The hatching date of each nestling was estimated by back‐calculating from the banding date and expressed as Julian date (Julian day number 1 assigned to January 1). Some breeders from Andalusia (*n* = 14), the Canary Islands (*n* = 80), and Segovia (*n* = 7) were banded as nestlings, which allowed us to know their identity and age. It is worth mentioning that there is a good age representation among breeders, with individuals ranging from 4 to 19 and 20 years old (a female and a male, respectively, which were still alive in the 2021 breeding season). Ages of breeding males and females were included in the analyses not only as their raw ages (in years) but also as categorical variables following Badia‐Boher et al. (2019; subadults: individuals younger than 6 years old, adults: 6–15 years old, and old adults: older than 15 years old) and Sanz‐Aguilar et al. (2017; young adults: 7 years old or less, and old adults: 8 years old or more). As the identity of the breeders was only available for a proportion of nestlings, we used subsets of data for each region available (peninsular Spain and Canary Islands; Table 1) to explore the effect of male and female age on offspring sex ratio separately, controlling for the effect of parental identity on nestling sex by including male or female identity as a random factor. Food availability was assessed taking into account changes resulting from the implementation of the European sanitary guidelines after the outbreak of the Bovine Spongiform Encephalopathy (i.e., the mad‐cow crisis) in the early 2000s, which limited the amount of livestock carcasses available to vultures and other scavengers (Almaraz et al., 2022; Donázar, Margalida, Carrete, & Sánchez‐Zapata, 2009). Thus, following Blanco (2014), food availability was included as a fixed factor with three levels corresponding to the main stages of the mad‐cow crisis, namely (i) prerestrictive period (1990–2001), before the mad‐cow crisis, characterized by a high availability of livestock carcasses in the field for vultures; (ii) restrictive period (2002–2011), during the mad‐cow crisis, when the abandonment of livestock carcasses was limited by new sanitary regulations (i.e., CE 1774/2002) and there was a severe shortage of food for vultures (around 80% of the livestock biomass available before this period was removed; Donázar, Margalida, & Campión, 2009); and (iii) postrestrictive period (2012 onwards), when the use of livestock carcasses for scavengers became more flexible (i.e., CE 142/2011 and RD 1632/2011) and food availability increased gradually (Almaraz et al., 2022; Morales‐Reyes et al., 2017). “Year” and “territory” were included as random factors in all models to avoid pseudoreplication. Collinearity between continuous variables was checked using the variance inflation factor (VIF). Bonferroni post hoc tests were used to compare the levels of each variable with a significant effect on nestling sex (package lsmeans; Lenth, 2016). Finally, using data from the Canary Islands, where monitoring has been much more intensive (Badia‐Boher et al., 2019), we calculated the repeatability in the probability that a breeding female (of known identity), pair or trio reared a male offspring across years as an indicator of the existence of a bias in the offspring sex ratio in each of them. Repeatability analyses were performed using the package rptR (Stoffel et al., 2017). Complementarily, we explored the offspring sex sequences of breeding females, pairs and trios (with known identity) from the Canary Islands that raised at least two nestlings of known sex (*n* = 63, *n* = 40 and *n* = 5, respectively) by means of two‐tailed binomial tests.

Insularity might mask the effect of other factors on offspring sex ratio, such as conspecific density and population trend. In the islands, Egyptian vultures are resident and the only obligate scavenger species, inhabit a limited space, and are well monitored over years. Therefore, using the annual number of breeding pairs as a proxy, we tested the effect of conspecific density on annual offspring sex ratio on both islands separately through GLMs (identity link function; Gaussian error distribution). Besides, focusing on the more extensive data from the Canary Islands, we assessed whether population trend could affect the annual offspring sex ratio (GLM; identity link function, Gaussian error distribution), using different time windows to estimate it. Specifically, we used seven variables, each one calculated as the difference between the number of breeding pairs in each year and the number of breeding pairs (i) the previous year, (ii) 2 years earlier, (iii) 3 years earlier, (iv) 4 years earlier, (v) 5 years earlier, (vi) 6 years earlier, and (vii) 7 years earlier, to account for time lags in the effects of population trend on offspring sex ratio. The range of 7 years was selected considering the typical age of first reproduction in this species (Sanz‐Aguilar et al., 2017).

Model selection was based on the Akaike Information Criterion adjusted for small sample size (AICc; Burnham & Anderson, 2002). Within each set of models (which includes the null model but not models that did not converge), we calculated the ΔAICc (i.e., the difference between the AICc of model i and that of the best model), and the Akaike weight (*w*) of each model (Burnham & Anderson, 2002). All models obtained for each scale of analysis (i.e., subset of data) were built using the same amount of data to make their AICc comparable. Models within 2 AICc units of the best were considered as alternative (Burnham & Anderson, 2002) and were used to perform model averaging (package MuMIn; Barton, 2017). An effect received no, weak, or strong support when the 95% confidence interval strongly overlapped zero, barely overlapped zero, or did not overlap zero, respectively. However, we will also discuss these alternative individual models to better understand the effects of our explanatory variables (Banner & Higgs, 2017). We used the DHARMa package (Hartig, 2022) to assess the fit of the final models. DHARMa employed a simulation‐based approach to create standardized residuals (values between 0 and 1) for fitted (generalized) linear (mixed) models and to test the significance of the dispersion parameter, zero‐inflation, and goodness‐of‐fit of the model (H0: fitted model fits the data well). Statistical analyses were performed in RStudio 4.1.2 (RStudioTeam, 2021).

## 3 RESULTS

**FIGURE 2:**
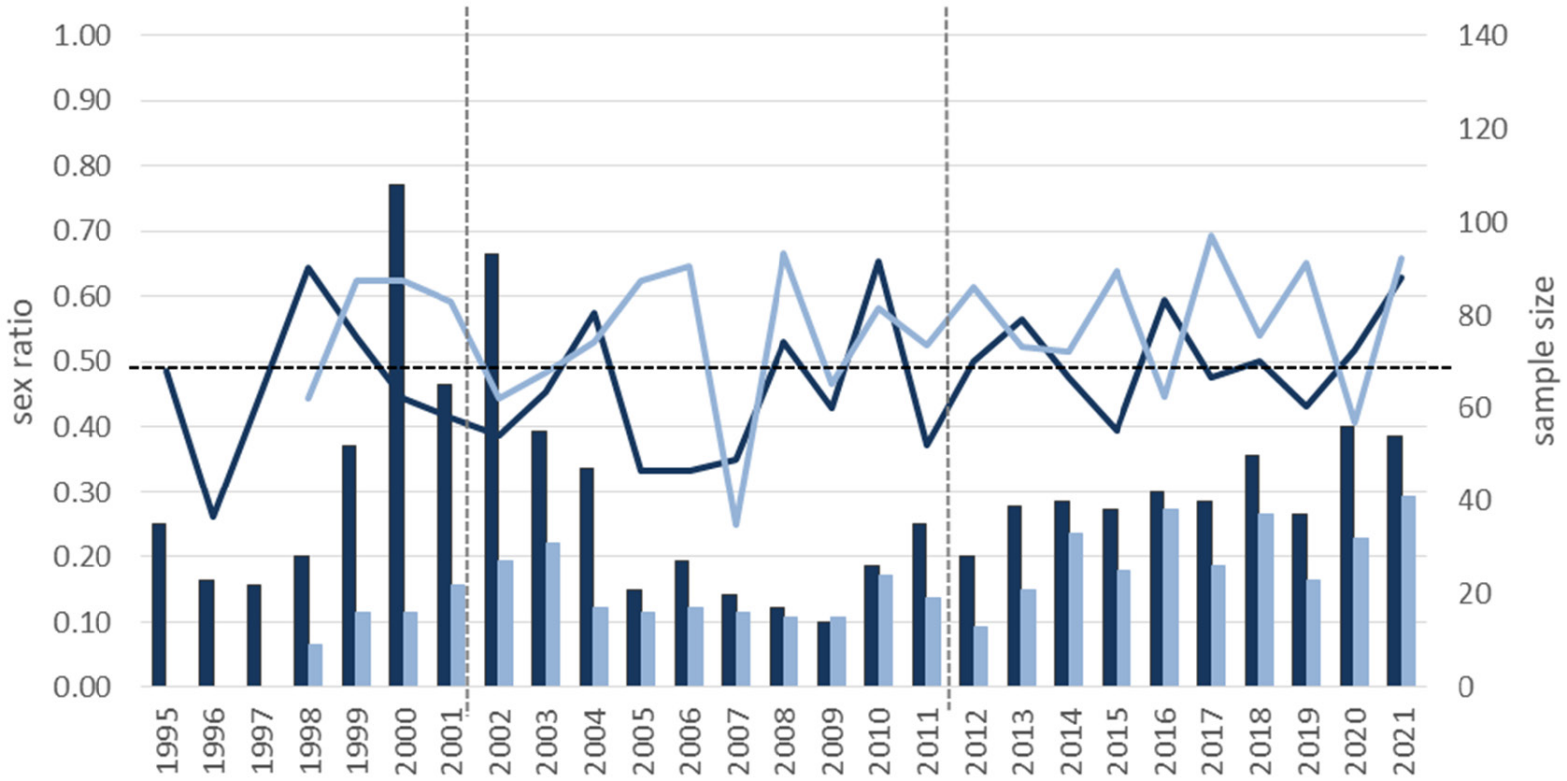
Secondary sex ratio (i.e., proportion of males over the total number of nestlings sexed per year; solid line) of Egyptian vultures for peninsular Spain (dark blue) and the islands (light blue), over the study years (1995–2021). Bars show sample sizes. Vertical dashed lines divide the main stages of the mad‐cow crisis (before, during, and after; see Section 2 for details). The horizontal dashed line marks a paired (0.5) sex ratio.

The overall sex ratio of Egyptian vulture nestlings was 1:1 (*n* = 1661; 829 males and 832 females). However, it was slightly female‐biased in peninsular Spain compared with the Canary Islands, where it was significantly male‐biased (Table 2, Figure 2; see Figure S1 for all histograms).

**TABLE 2:** Number of male and female Egyptian vulture nestlings and secondary sex ratio in the different sampled regions of Spain (mainland and islands).

Region (study period)	*n*	Males	Females	Sex ratio	2.5% CI	97.5% CI
Peninsular Spain	1112	528	584	**0.47**	0.48	0.52
Andalusia (2000–2021)	268	123	145	0.46	0.44	0.56
Aragon (1995–2004)	237	113	124	0.48	0.44	0.56
Navarra (1995–2004/2010–2021)	425	208	217	0.49	0.46	0.54
Segovia (2004–2021)	182	84	98	0.46	0.43	0.57
Islands	549	301	248	**0.55**	0.46	0.54
Canary Islands (1998–2021)	499	275	224	**0.55**	0.46	0.54
Balearic Islands (1998–2003)	50	26	24	0.52	0.36	0.62
All	1661	829	832	0.50	0.48	0.52

*Note*: Sex ratios are expressed as the proportion of males over the total number of nestlings sexed. In bold, values of observed sex ratios outside the 95% confidence interval (CI) obtained in the simulations.

Pooling nestlings from peninsular Spain or from the islands, we found no significant differences (all *p* > .05) toward a particular sex in any year, with sex ratios fluctuating between 0.26–0.65 and between 0.25 and 0.69, respectively (Figure 2; see Table S1 for results of specific models testing for differences). This same pattern was observed for all nestlings grouped together (range 0.26–0.64).

In double broods, we found a significant bias in the Canary Islands toward two‐male broods rather than two‐female broods, in accordance with the male‐biased offspring sex ratio on these islands (Table 3). No biased patterns were found for the other regions, neither when comparing mixed broods with a first‐hatched male and mixed broods with a first‐hatched female (Table 3). Also, the number of single‐sex broods was not significantly different from that of different sexes in any region (Table 3).

**TABLE 3:** Frequency of sex combinations in double broods of the different regions sampled (*n* = 391): two males (mm), two females (ff), or both sexes, with the male (mf) or the female (fm) being the older nestling.

Region	mm	ff	*p*‐Value	mf	fm	*p*‐Value	Single sex	Mixed sex	*p*‐Value
Peninsular Spain	77	84	.6364	71	83	.3755	161	154	.7354
Andalusia	12	15	.7011	11	21	.1102	27	32	.6029
Aragon	14	22	.2430	15	22	.3240	36	37	1.0000
Navarra	38	34	.7239	31	24	.4188	72	55	.1554
Segovia	13	13	1.0000	14	16	.8555	26	30	.6889
Islands	31	13	**.0096**						
Canary Isl.	27	12	**.0237**	20	12	.2153	39	32	.4767
Balearic Isl.	4	1	.3750	–	–	–	–	–	–
All	108	97	.4850	91	95	.8260	205	186	.3627

*Note*: “Single sex” broods consist of two males or two females, while “mixed sex” broods correspond to broods formed by one male and one female, regardless of their hatching order. *p*‐values correspond to the two‐sided binomial tests. In bold, significant *p*‐values.

### 3.1 Effects of parental age on offspring sex ratio

The age of the breeding individuals (female: range = 4–19 years old, *n* = 44; male: range = 4–20 years old, *n* = 57) did not affect the probability of a nestling being a male, neither when data from peninsular areas were considered nor when only data from the Canary Islands were used (Table S2). Models including other proxies of parental age (categorical) yielded similar results (Table S3).

### 3.2 Effects of individual and environmental factors on offspring sex ratio

All candidate models included the effect of insularity on the probability of an Egyptian vulture nestling being a male (Table 4). Probability was higher in the Canary Islands (55.1% males, 44.9% females; *n* = 499) compared with the mainland (47.5% males, 52.5% females; *n* = 1112). Offspring sex ratio was also male‐biased after the mad‐cow crisis, when livestock carcasses were again progressively abandoned in the field (53.3% males, 46.7% females; *n* = 713), compared with the other two periods (before: 47.8% males, 52.2% females; *n* = 372; during: 46.6% males, 53.4% females; *n* = 526). However, *post hoc* tests were only marginally significant for the comparison between the periods after and during the crisis (*p* = .0585). Brood size and hatching date were also included in the set of alternative models (ΔAICc < 2), although the 95% CI of their estimates overlapped zero (Table 4; see Table S5 for model outputs).

**TABLE 4:** Alternative models (ΔAICc < 2) obtained to assess the effects of hatching date (hatching date), region (region), insularity (insularity), food availability (mad‐cows), brood size (brood size), and hatching order (order) on the probability of an Egyptian vulture nestling being a male (*n* = 1611).

Model selection				
Model	df	AICc	ΔAICc	*w*
Insularity + brood size	5	2230.89	0.00	0.19
Insularity	4	2232.42	1.53	0.09
Insularity + mad‐cows	6	2232.66	1.76	0.08
Insularity + mad‐cows + brood size + hatching date	8	2232.67	1.78	0.08
Insularity + brood size + hatching date	6	2232.73	1.84	0.08

Model averaging				
Variable	Estimate	SE	2.5% CI	97.5% CI
Intercept	−0.21	0.11	−0.43	0.01
Brood size (2)	0.20	0.11	−0.01	0.40
**Insularity (island)**	**0.32**	**0.13**	**0.06**	**0.57**
**Mad‐cows (post)**	**0.25**	**0.12**	**0.02**	**0.48**
Mad‐cows (pre)	0.11	0.14	−0.17	0.38
Hatching date	−0.03	0.07	−0.16	0.10

*Note*: Estimates, standard errors (SE), and 95% confidence intervals (CI) were obtained after model averaging. All models were run including year and territory as random terms. The null model was included in our set of models. In bold, significant effects (i.e., the 95% CI does not overlap zero). All models run are shown in Table S4. Model fits are shown in Figure S2. Nestlings from the Balearic Islands were excluded from this analysis since data on hatching date and order were unavailable.

Abbreviations: AICc, Akaike information criterion corrected for small sample sizes; df, degrees of freedom; *w*, Akaike weight; ΔAICc, difference between the AICc of model *i* and that of the best model (i.e., the model with the lowest AICc).

#### 3.2.1 Peninsular Spain

In peninsular Spain, we found no differences among natal regions in the secondary sex ratio but a higher probability of a nestling being a male in the period after the mad‐cow crisis (Table 5). Nevertheless, post hoc tests showed no significant differences between the three periods considered, suggesting a very weak effect. Brood size, hatching date and hatching order were included in alternative models (ΔAICc < 2) but their estimates were not different from zero (Table 5).

**TABLE 5:** Alternative models (ΔAICc < 2) obtained to assess the effects of hatching date (hatching date), region (region), food availability (mad‐cows), brood size (brood size), and hatching order (order) on the probability of an Egyptian vulture nestling being a male in peninsular Spain (*n* = 1112).

Model selection				
Model	df	AICc	ΔAICc	*w*
Null	3	1543.78	0.00	0.17
Mad‐cows	5	1544.15	0.38	0.14
Brood size	4	1544.17	0.40	0.14
Mad‐cows + brood size	6	1544.42	0.64	0.12
Order	5	1545.66	1.88	0.07
Hatching date	4	1545.74	1.96	0.06

Model averaging				
Variable	Estimate	SE	2.5% CI	97.5% CI
Intercept	−0.19	0.13	−0.45	0.06
**Mad‐cows (post)**	**0.30**	**0.15**	**0.01**	**0.58**
Mad‐cows (pre)	0.09	0.16	−0.22	0.39
Brood size (2)	0.16	0.12	−0.08	0.40
Order (1)	0.10	0.15	−0.19	0.39
Order (2)	0.21	0.15	−0.07	0.50
Hatching date	0.01	0.06	−0.10	0.13

*Note*: Estimates, standard errors (SE), and 95% confidence intervals (CI) were obtained after model averaging. All models were run including year and territory as random terms. The null model was included in our set of models. In bold, significant effects (i.e., the 95% CI does not overlap zero). All models run are shown in Table S6.

Abbreviations: AICc, Akaike information criterion corrected for small sample sizes; df, degrees of freedom; *w*, Akaike weight; ΔAICc, difference between the AICc of model *i* and that of the best model (i.e., the model with the lowest AICc).

When analyzing only first‐hatched nestlings from double broods in peninsular Spain, the probability of a nestling being a male was higher after the mad‐cow crisis (55.6% males, 44.4% females; *n* = 124; Table 6; see Table S7 for model outputs). Additionally, the nonsignificant *post hoc* tests suggest a very small effect of the natal region. Models obtained considering only the second‐hatched nestlings from double broods showed no effects of any variable (Table 6).

**TABLE 6:** Models obtained to assess the effects of hatching date (hatching date), region (region) and food availability (mad‐cows) on the probability of an Egyptian vulture nestling being a male in peninsular Spain, (a) considering only first‐hatched nestlings from double broods (*n* = 324), and (b) considering only second‐hatched nestlings from double broods (*n* = 318).

Model selection				
Model	df	AICc	ΔAICc	*w*
(a) First‐hatched nestlings from double broods				
Mad‐cows	5	451.92	0.00	0.32
Mad‐cows + region	8	453.19	1.28	0.17
Region	6	453.82	1.90	0.12
Null	3	453.84	1.92	0.12
Mad‐cows + hatching date	6	453.93	2.01	0.12
Mad‐cows + region + hatching date	9	455.31	3.39	0.06
Hatching date	4	455.60	3.68	0.05
Region + hatching date	7	455.90	3.98	0.04
(b) Second‐hatched nestlings from double broods				
Null	3	446.87	0.00	0.40
Mad‐cows	5	448.04	1.17	0.22
Hatching date	4	448.21	1.35	0.20
Hatching date + mad‐cows	6	449.25	2.38	0.12
Region	6	452.06	5.19	0.03
Hatching date + region	7	453.44	6.57	0.01
Mad‐cows + region	8	453.98	7.11	0.01
Hatching date + mad‐cows + region	9	455.33	8.46	0.01

Model averaging				
Variable	Estimate	SE	2.5% CI	97.5% CI
(a)				
Intercept	−0.50	0.35	−1.19	0.19
**Mad‐cows (post)**	**0.70**	**0.30**	**0.11**	**1.28**
Mad‐cows (pre)	0.30	0.32	−0.33	0.93
Region (Aragon)	0.25	0.43	−0.60	1.10
**Region (Navarra)**	**0.74**	**0.36**	**0.03**	**1.45**
Region (Segovia)	0.34	0.41	−0.47	1.15
(b)				
Intercept	0.06	0.16	−0.24	0.37
Mad‐cows (post)	−0.02	0.28	−0.56	0.53
Mad‐cows (pre)	−0.42	0.29	−0.98	0.14
Hatching date	−0.09	0.11	−0.32	0.13

*Note*: Estimates, standard errors (SE), and 95% confidence intervals (CI) were obtained after model averaging. All models were run including year and territory as random terms. The null model was included in our set of models. In bold, significant effects (i.e., the 95% CI does not overlap zero).

Abbreviations: AICc, Akaike information criterion corrected for small sample sizes; df, degrees of freedom; *w*, Akaike weight; ΔAICc, difference between the AICc of model *i* and that of the best model (i.e., the model with the lowest AICc).

#### 3.2.2 Islands

On the Balearic Islands, no variable showed a significant relationship with the probability of a nestling being a male (Table S8). Brood size was included in one of the alternative models (ΔAICc < 2), but the 95% CI of its estimate overlapped zero (Table S8).

On the Canary Islands, where the offspring sex ratio was biased toward males, we found a higher probability of a nestling being a male in the first nestling of double broods compared with single nestlings (Table 7). However, *post hoc* tests were not significant (*p* = .1255). The other variables considered, some of them included in the alternative models (ΔAICc < 2), did not contribute to explain nestling sex (Table 7).

**TABLE 7:** Alternative models (ΔAICc < 2) obtained to assess the effects of hatching date (hatching date), food availability (mad‐cows), brood size (brood size), hatching order (order), and type of breeding unit (unit) on the probability of an Egyptian vulture nestling being a male in the Canary Islands (*n* = 499).

Model selection				
Model	df	AICc	ΔAICc	*w*
Brood size	4	691.92	0.00	0.10
Order	5	692.04	0.12	0.10
Null	3	692.27	0.36	0.09
Unit	5	692.76	0.85	0.07
Brood size + unit	6	692.76	0.85	0.07
Order + unit	7	692.88	0.97	0.06
Hatching date	4	693.02	1.11	0.06
Brood size + hatching date	5	693.08	1.16	0.06
Unit + hatching date	6	693.21	1.30	0.05
Order + hatching date	6	693.44	1.52	0.05
Brood size + hatching date + unit	7	693.65	1.73	0.04

Model averaging				
Variable	Estimate	SE	2.5% CI	97.5% CI
Intercept	0.14	0.13	−0.10	0.39
Brood size (2)	0.30	0.21	−0.11	0.71
**Order (1)**	**0.55**	**0.28**	**0.00**	**1.10**
Order (2)	0.07	0.27	−0.45	0.60
Hatching date	−0.10	0.10	−0.29	0.09
Unit (pair)	−0.10	0.20	−0.50	0.30
Unit (trio)	0.74	0.46	−0.16	1.64

*Note*: Estimates, standard errors (SE), and 95% confidence intervals (CI) were obtained after model averaging. All models were run including year and territory as random terms. The null model was included in our set of models. In bold, significant effects (i.e., the 95% CI does not overlap zero). All models run are shown in Table S9.

Abbreviations: AICc, Akaike information criterion corrected for small sample sizes; df, degrees of freedom; *w*, Akaike weight; ΔAICc, difference between the AICc of model *i* and that of the best model (i.e., the model with the lowest AICc).

Neither conspecific density, measured as the annual number of breeding pairs, nor the different population trend estimates used for the Canary Islands, influenced the offspring sex ratio (models with ΔAICc > 2; Tables S10 and S11, respectively).

### 3.3 Sex ratio and sex sequences in breeding females, pairs and trios from the Canary Islands

Neither females nor breeding pairs with known identity from the Canary Islands showed a clear tendency to systematically rear male offspring (*r* = 0.00 ± 0.01, 95% CI 0.00–0.04, and *r* = 0.00 ± 0.02, 95% CI 0.00–0.06, respectively). Nevertheless, from the more detailed information available from some of those breeding units, there were slightly more sequences of four or more consecutive male nestlings (*n* = 11 breeding females) than of four or more consecutive female nestlings (*n* = 3 breeding females; binomial test: *p* = .0574; Table S12).

As with females and breeding pairs, there was no repeatability in offspring sex for trios with known identity from the Canary Islands (*r* = 0.23 ± 2.63, 95% CI 0.00–0.81), although the sample size was very small. When we analyzed the more detailed information available from some of those breeding trios, we found that one female–female–male trio was associated with a strong bias, raising only males (*n* = 9) over the 9 years sampled. This trio was the only known breeding unit in our database to produce a significantly male‐biased offspring sex ratio (binomial test: *p* = .0039).

## 4 DISCUSSION

Overall, the secondary sex ratio in nestling Egyptian vultures did not differ significantly from parity, as would be expected in a species with slight sexual dimorphism and a relatively balanced role in parental duties (Donázar, 1993; Morant et al., 2019). This result, obtained from a database comprised of hundreds of individuals monitored in different areas over ca. 30 years, is consistent with that found for this species using subsamples of this dataset (Grande, 2006; Sanz‐Aguilar et al., 2017) and in other species of vultures from Europe (Bosé et al., 2007; Davidovic et al., 2022; Gómez‐López et al., 2022; López‐López et al., 2011; Villegas et al., 2004) and Asia (Arshad et al., 2009). We also found that the offspring sex ratio of the Egyptian vulture remained stable at 1:1 over the years, similar to studies with the Griffon Vulture (Gómez‐López et al., 2022) and the Cinereous Vulture (Villegas et al., 2004) in regions of peninsular Spain. However, offspring sex ratio was male‐biased in the Canary Islands, while a slight trend toward females was observed in peninsular Spain. These opposite patterns could be associated with factors linked to the island syndrome (see Covas, 2016), which has already been documented in this species (Donázar et al., 2002).

There are several factors linked with insularity that can be taken into consideration here. First, the age composition of the breeders varies between mainland and island populations. The percentage of subadult breeders is essentially zero in the Peninsula (Ferrer et al., 2011), but has been proved to be relatively high (ca. 10%) and constant in the Canary Islands over our study years (Donázar et al., 2002; J. A. Donázar, unpublished data). As younger breeders are also less experienced and display poorer foraging abilities, they typically overproduce the less costly sex, males in this case, which could be unbalancing the overall offspring sex ratio in the islands. Although we did not find an apparent effect of the age of the breeders on sex ratio, it must be noted that our data only constitute a sample of the breeding population, so there may still be an effect operating at the population level that we have been unable to detect and that would become evident with increasing sample size. Second, survival of juveniles (<1 year) is higher in the Canary and the Balearic Islands (*ϕ* ≈ 0.90; Badia‐Boher et al., 2019; Donázar et al., 2002; Sanz‐Aguilar, De Pablo, & Donázar, 2015) than in continental Spain (*ϕ* ≈ 0.70; Grande et al., 2009; Sanz‐Aguilar, Sánchez‐Zapata, et al., 2015) and Europe (*ϕ* ≈ 0.30–0.70; Lieury et al., 2015; Oppel, Saravia, et al., 2021), and it is very similar to that of subadults and adults, a common pattern found in nonmigratory raptor populations from islands (Badia‐Boher et al., 2019; Sanz‐Aguilar, De Pablo, & Donázar, 2015). The low mortality reported in these populations probably comes from their sedentary behavior, which avoids high migration costs (Oppel, Arkumarev, et al., 2021; Sanz‐Aguilar, De Pablo, & Donázar, 2015), but is also a consequence of intense management actions, mainly through supplementary feeding and severe control and correction of human‐related mortality factors, like poisoning, electrocution, and collision with power lines (Badia‐Boher et al., 2019; Donázar et al., 2002; Sanz‐Aguilar, De Pablo, & Donázar, 2015). Although Egyptian vulture survival according to sex has not been studied in the islands, some populations from peninsular Spain show differential survival depending on both age and sex, with breeding females and young breeding males displaying lower survival rates than old breeding males (Sanz‐Aguilar et al., 2017). Thus, if the adult sex ratio remains at the expected 1:1 in peninsular Spain (Grande, 2006), the slight female bias among the offspring would be later compensated for by a sex‐biased mortality toward breeding females (Sanz‐Aguilar et al., 2017). On the contrary, if higher rates of breeding female mortality were true for the islands in addition to a male‐biased offspring sex ratio, we might expect a reduction in the amount of breeding females and an increase in the number of polyandrous trios. However, this pattern is probably not valid for the Canary Islands. Even though the offspring sex ratio is male‐biased, the adult sex ratio has been reported to be around 1:1 (Gangoso, 2006; J. A. Donázar, unpublished data), just opposite to what Donald (2007) predicted for most bird species. Since the adult sex ratio is apparently unbiased, mortality rates could be male‐biased between the nestling and the adult stage, maybe due to a more intense territory prospection and large‐scale movements involving greater exposure to risks, by immature males compared with females (Sanz‐Aguilar et al., 2017; Van Overveld et al., 2018). One of those risks is poisoning by lead ammunition, which is less frequent among females from the Canary Islands since they tend to feed at supplementary feeding stations rather than on farms or over natural areas (Gangoso et al., 2009; García‐Heras et al., 2013; Van Overveld et al., 2018). Raw data from the Canary Islands show that the proportion of recovered dead immature vs adult individuals was indeed higher for males (64.3%) than for females (52.9%) (J. A. Badia‐Boher & A. Sanz‐Aguilar, unpublished data). This male‐biased mortality among immatures could be ultimately compensating for offspring biases, so that the adult sex ratio would not reflect early‐biased sex ratios.

Food availability might also differ between the mainland and the islands, but it is difficult to quantify appropriately. On the one hand, the mad‐cow crisis, which led to a reduction in the availability of livestock carcasses, caused strong declines in the productivity and the reproductive success of several vulture populations (Almaraz et al., 2022; Iñigo & Atienza, 2007). However, it is a very general proxy as it refers to livestock availability only. Furthermore, since sanitary regulations were unequally applied outside of and throughout the Spanish territory (Arrondo et al., 2018; López‐Bao & Margalida, 2018), the availability of livestock carcasses varied considerably between years in more restrictive regions (e.g., Andalusia and Navarra), while more permissive areas held high levels of livestock carrion over time (e.g., Aragon and Canary Islands; Donázar, Cortés‐Avizanda, et al., 2020; García‐Alfonso et al., 2020). On the other hand, Egyptian vultures are not only dependent on livestock carcasses but also rely on wild animals, such as rabbits, birds or insects, which can represent more than 50% of their diverse diet depending on the region (Donázar & Ceballos, 1988; Margalida et al., 2011; Medina, 1999). Hence, long‐term changes in wild prey abundance and distribution caused by natural or anthropogenic factors might influence the amount, type, distribution, predictability, and availability of food for these facultative scavengers (Blanco, 2014; Donázar, Barbosa, et al., 2020). Consequently, depending on regional and local trophic variability, populations are likely to be affected differently (Donázar, Cortés‐Avizanda, et al., 2020; Margalida et al., 2011). However, although the amount of livestock carcasses is apparently high in the eastern Canary Islands (Donázar et al., 2002), its conditions of extreme aridity reduce the abundance of small, wild prey available for vultures (J. A. Donázar, unpublished data), so raising the less costly males could be a better option for breeders than raising females in this population (Dzus et al., 1996; Wiebe & Bortolotti, 1992). In addition to spatial food asymmetries, there is considerable variability regarding vulture population trends and conspecific densities among regions and over time (Del Moral & Molina, 2018), which might also be influencing sex ratio in different ways. For instance, the Canary Islands population is growing and displays a high conspecific density (Badia‐Boher et al., 2019), partly due to the limited dispersal and the isolated character of the islands, while the mainland populations considered here are declining and their densities vary considerably (Serrano et al., 2021). Thus, density dependence can negatively affect parameters such as body mass (Donázar, Barbosa, et al., 2020) and productivity (Carrete, Donázar, & Margalida, 2006) but it could also influence offspring sex ratio. We were unable to obtain suitable measures of conspecific density and population trends in mainland populations that would allow us to detect a potential effect of these parameters on offspring sex ratio. Nevertheless, given that neither of these two connected variables influenced offspring sex ratio in the Canary Islands, we suggest that the traits related to the island syndrome, other than conspecific density or population trend, are the main factors causing the male bias in this study area.

Siblicide by the first‐hatched nestling of the second‐hatched through direct aggression or food monopolization has been reported in the Egyptian vulture (Birdlife Israel, 2021; Brown et al., 1982), but it is not common (Kumar et al., 2020; Redondo et al., 2019; Yordanov et al., 2021). Dzus et al. (1996) found a relationship between food availability, hatching order, and nestling sex in the Bald Eagle, a siblicidal species, and proposed that mixed broods where the female hatches first are more likely to be found in good years, when food competition is low, siblicide is rarer and both nestlings can be raised (Dzus et al., 1996; Uller, 2006). Besides, studies with nonsiblicidal species conclude that, since females, the larger sex, are more susceptible to food stress (Clutton‐Brock, 1986), they often hatch first in the brood under adverse environmental conditions, so that they get the preferential parental investment and survive (Carranza, 2004). Although our results on the sex ratio of first‐hatched nestlings from peninsular Spain do not match our expectations, they are consistent with the hypothesis proposed by Carranza (2004): Food limitations could be biasing first‐hatched nestlings toward females during low‐resource periods (mad‐cow crisis), which would survive thanks to a better parental investment, while high food availability might bias first‐hatched nestlings toward either sex after the crisis (males) or before it (females), when both sexes would be raised equally and no or little aggression would occur. However, no direct effect of hatching order on sex ratio was detected in the analyses, as reported in some other raptors (Hörnfeldt et al., 2000; McDonald et al., 2005; Rutz, 2012). Additionally, despite relevant variation in other factors such as hatching date or parental age, the rest of the variables studied did not appear to have an effect on offspring sex ratio. Brood size in particular is not associated with sex ratio biases in other studies with raptor nestlings (Byholm et al., 2002; Rutz, 2012; Wiebe & Bortolotti, 1992), and as variation in brood size is minimal in the Egyptian vulture (one or two nestlings), the effect of brood size on sex ratio is likely more difficult to be found in this species. The apparent lack of influence of other factors on the offspring sex ratio could also be due to the fact that the costs of rearing each sex might not be very different in the Egyptian vulture.

Regular monitoring of Egyptian vulture breeding units in the Canary Islands allowed us to assess individual effects, that is, whether particular pairs, trios or females are more prone to produce offspring of a particular sex, consecutively or not (see Heinsohn et al., 1997), but we found little evidence of any pattern. One trio raised nine male nestlings in a row so trios should be especially monitored in the following years to detect possible general patterns. Also, the prevalence of at least four consecutive male nestlings was higher than that of at least four consecutive female nestlings, as expected for the overall male‐biased sex ratio in the population. No difference was observed between the amount of same‐sex broods and mixed‐sex broods in any of our regions of study. Furthermore, it must be noted that studying the process of sex allocation is very complex. Multiple factors like those addressed in the present research are often interrelated, and it is very hard to identify which environmental factors are causing biases through a direct or indirect effect on offspring sex ratio at the brood, subpopulation or population level in the different species. In addition, the amount of parental contribution to the process by directly biasing sex ratio as a response to the environmental changes or by their individual condition makes the subject even more complicated to study.

Here, we combined a high number of Egyptian vulture individuals from some of the most important breeding regions for this species in both continental and insular Spain, but we acknowledge certain limitations. There was a different number of nestlings sampled each year (between 22 and 124) and region (between 50 and 499). Particularly, the Balearic Islands population could not be completely assessed since sample size was very small, especially compared with the Canary Islands. Some explanatory variables were not available for all nestlings (e.g., hatching date, hatching order, and parental age), either due to differences in the data collected by each monitoring program or to the high sampling effort needed to obtain certain information in the field. To further study the effects of parental age on offspring sex ratio, which could not be properly assessed in peninsular Spain, additional data should be collected in the future. Differences in the type of trio (two males and one female, or two females and one male) might also affect offspring sex ratio, but since Egyptian vulture trios are relatively rare, the small size of the resulting sample prevented us from assessing this properly. Although we evaluated the effect of the mad‐cow crisis as a surrogate of food availability, other food variables should be considered in future studies. Unfortunately, we did not have information concerning the nonbreeding fraction of the island populations, which we acknowledge could have some relevance in conspecific density analyses. Nevertheless, our study shows that insularity is an important predictor of offspring sex ratio of the Egyptian vulture, probably through processes that affect island and mainland populations differentially. In cases when immature mortality is not biased toward males, a male‐biased offspring sex ratio should be monitored with caution, especially in an isolated subspecies like *N. p. majorensis* from the Canary Islands, as it could have important consequences for the future dynamics and viability of the population. Our research also contributes to sex allocation theory by investigating whether sex ratio deviations from parity are possible as a response to changing environments, either through parental manipulation or through sex‐biased mortality by environmental and social constraints, here tested by proxies. Getting to know the multiple and complexly interrelated factors involved in these deviations is essential for understanding underlying issues in the ecology and life history of raptors as well as helping in the development and application of conservation practices in threatened species which, like the Egyptian vulture, are especially vulnerable in a global change scenario.

## AUTHOR CONTRIBUTIONS

**Guillermo Gómez‐López:** Conceptualization (equal); data curation (equal); formal analysis (equal); writing – original draft (lead). **Ana Sanz‐Aguilar:** Conceptualization (equal); writing – review and editing (equal). **Martina Carrete:** Conceptualization (equal); formal analysis (equal); writing – review and editing (equal). **Eneko Arrondo:** Resources (equal); writing – review and editing (equal). **José Ramón Benítez:** Data curation (equal); resources (equal); writing – review and editing (equal). **Oolga Ceballos:** Data curation (equal); resources (equal); writing – review and editing (equal). **Ainara Cortés‐Avizanda:** Data curation (equal); resources (equal); writing – review and editing (equal). **Félix de Pablo:** Data curation (equal); resources (equal); writing – review and editing (equal). **José Antonio Donázar:** Data curation (equal); resources (equal); writing – review and editing (equal). **Óscar Frías:** Resources (equal); writing – review and editing (equal). **Laura Gangoso:** Resources (equal); writing – review and editing (equal). **Marina García‐Alfonso:** Resources (equal); writing – review and editing (equal). **José Luis González:** Resources (equal); writing – review and editing (equal). **Juan Manuel Grande:** Resources (equal); writing – review and editing (equal). **David Serrano:** Resources (equal); writing – review and editing (equal). **José Luis Tella:** Resources (equal); writing – review and editing (equal). **Guillermo Blanco:** Conceptualization (equal); data curation (equal); writing – review and editing (equal).

## CONFLICT OF INTEREST STATEMENT

The authors declare no conflict of interest.

## Supporting information


Data S1

## Data Availability

We have uploaded our dataset to a different repository than the one proposed in the manuscript. The section should be changed to: Data are available at Zenodo (https://doi.org/10.5281/zenodo.8147836).

## References

[ece310371-bib-0001] Agudo, R. , Carrete, M. , Alcaide, M. , Rico, C. , Hiraldo, F. , & Donázar, J. A. (2012). Genetic diversity at neutral and adaptive loci determines individual fitness in a long‐lived territorial bird. Proceedings of the Royal Society B: Biological Sciences, 279(1741), 3241–3249. 10.1098/rspb.2011.2606 PMC3385713 22553093

[ece310371-bib-0002] Almaraz, P. , Martínez, F. , Morales‐Reyes, Z. , Sánchez‐Zapata, J. A. , & Blanco, G. (2022). Long‐term demographic dynamics of a keystone scavenger disrupted by human‐induced shifts in food availability. Ecological Applications, 32, e2579. 10.1002/eap.2579 35279905

[ece310371-bib-0003] Alonso‐Alvarez, C. (2006). Manipulation of primary sex‐ratio: An updated review. Avian and Poultry Biology Reviews, 17(1), 1–20. 10.3184/147020606783437930

[ece310371-bib-0004] Anderson, D. , Reeve, J. , Gomez, J. , Weathers, W. , Hutson, S. , Cunningham, H. , & Bird, D. (1993). Sexual size dimorphism and food requirements of nestling birds. Canadian Journal of Zoology, 71, 2541–2545.

[ece310371-bib-0005] Arrondo, E. , Moleón, M. , Cortés‐Avizanda, A. , Jiménez, J. , Beja, P. , Sánchez‐Zapata, J. A. , & Donázar, J. A. (2018). Invisible barriers: Differential sanitary regulations constrain vulture movements across country borders. Biological Conservation, 219, 46–52. 10.1016/j.biocon.2017.12.039

[ece310371-bib-0006] Arroyo, B. E. (2002). Fledgling sex ratio variation and future reproduction probability in Montagu's harrier, *Circus pygargus* . Behavioral Ecology and Sociobiology, 52(2), 109–116. 10.1007/s00265-002-0496-9

[ece310371-bib-0007] Arshad, M. , Chaudhary, M. J. I. , & Wink, M. (2009). High mortality and sex ratio imbalance in a critically declining oriental white‐backed vulture population (*Gyps bengalensis*) in Pakistan. Journal of Ornithology, 150, 495–503. 10.1007/s10336-008-0368-9

[ece310371-bib-0008] Badia‐Boher, J. A. , Sanz‐Aguilar, A. , de la Riva, M. , Gangoso, L. , García‐Alfonso, M. , Van Overveld, T. , Luzardo, O. P. , Suarez‐Pérez, A. , & Donázar, J. A. (2019). Evaluating European LIFE conservation projects: Improvements in survival of an endangered vulture. Journal of Applied Ecology, 56, 1210–1219. 10.1111/1365-2664.13350

[ece310371-bib-0009] Banner, K. M. , & Higgs, M. D. (2017). Considerations for assessing model averaging of regression coefficients. Ecological Applications, 27, 78–93. 10.1002/eap.1419 27874997

[ece310371-bib-0010] Barton, K. (2017). MuMIn: Multi‐Model Inference (R package) . https://cran.r‐project.org/package=MuMIn

[ece310371-bib-0011] Bednarz, J. C. , & Hayden, T. J. (1991). Skewed brood sex ratio and sex‐biased hatching sequence in Harris's hawks. American Naturalist, 137(1), 116–132. 10.1086/285149

[ece310371-bib-0012] Bertran, J. , & Margalida, A. (2002). Social organization of a trio of bearded vultures (*Gypaetus barbatus*): Sexual and parental roles. Journal of Raptor Research, 36(1), 66–70.

[ece310371-bib-0013] Birdlife International . (2020). *Neophron percnopterus*. The IUCN Red List of Threatened Species 2020: E.T22695180A166295484. https://www.iucnredlist.org/species/22695180/166295484

[ece310371-bib-0014] Birdlife International . (2021). *Neophron percnopterus*. The IUCN Red List of Threatened Species 2021: E.T22695180A205187871. https://www.iucnredlist.org/species/22695180/205187871

[ece310371-bib-0015] Birdlife Israel . (2021). End of the Egyptians breeding season . https://www.birds.org.il/en/blog/id/25/1540

[ece310371-bib-0016] Blanco, G. (2014). Can livestock carrion availability influence diet of wintering red kites? Implications of sanitary policies in ecosystem services and conservation. Population Ecology, 56(4), 593–604. 10.1007/s10144-014-0445-2

[ece310371-bib-0017] Blanco, G. , Dávila, J. A. , Septiem, J. A. , Rodríguez, R. , & Martínez, F. (2002). Sex‐biased initial eggs favours sons in the slightly size‐dimorphic Scops owl (*Otus scops*). Biological Journal of the Linnean Society, 76(1), 1–7.

[ece310371-bib-0018] Blanco, G. , Martínez‐Padilla, J. , Dávila, J. A. , Serrano, D. , & Viñuela, J. (2003). First evidence of sex differences in the duration of avian embryonic period: Consequences for sibling competition in sexually dimorphic birds. Behavioral Ecology, 14(5), 702–706. 10.1093/beheco/arg049

[ece310371-bib-0019] Blanco, G. , Pais, J. L. , Fargallo, J. A. , Potti, J. , Lemus, J. A. , & Dávila, J. A. (2009). High proportion of non‐breeding individuals in an isolated red‐billed chough population on an oceanic Island (La Palma, Canary Islands). Ardeola, 56(2), 229–239.

[ece310371-bib-0020] Blank, J. L. , & Nolan, V. (1983). Offspring sex ratio in red‐winged blackbirds is dependent on maternal age. Proceedings of the National Academy of Sciences of the United States of America, 80(19), 6141–6145. 10.1073/pnas.80.19.6141 16593379 PMC534377

[ece310371-bib-0021] Blondel, J. (2000). Evolution and ecology of birds on islands: Trends and prospects. Vie et Milieu, 4, 205–220.

[ece310371-bib-0022] Bonal, R. , & Aparicio, J. M. (2008). Evidence of prey depletion around lesser kestrel *Falco naumanni* colonies and its short term negative consequences. Journal of Avian Biology, 39(2), 189–197. 10.1111/j.2008.0908-8857.04125.x

[ece310371-bib-0023] Bortolotti, G. R. (1986). Influence of sibling competition on nestling sex ratios of sexually dimorphic birds. American Naturalist, 127(4), 495–507.

[ece310371-bib-0024] Bosé, M. , Le Gouar, P. , Arthur, C. , Lambourdière, J. , Choisy, J. P. , Henriquet, S. , Lecuyer, P. , Richard, M. , Tessier, C. , & Sarrazin, F. (2007). Does sex matter in reintroduction of griffon vultures *Gyps fulvus*? Oryx, 41(4), 503–508. 10.1017/S0030605307000312

[ece310371-bib-0025] Bradbury, R. B. , & Blakey, J. K. (1998). Diet, maternal condition, and offspring sex ratio in the zebra finch, *Poephila guttata* . Proceedings of the Royal Society B: Biological Sciences, 265(1399), 895–899. 10.1098/rspb.1998.0375

[ece310371-bib-0026] Brouwer, L. , Tinbergen, J. M. , Both, C. , Bristol, R. , Richardson, D. S. , & Komdeur, J. (2009). Experimental evidence for density‐dependent reproduction in a cooperatively breeding passerine. Ecology, 90(3), 729–741. 19341143 10.1890/07-1437.1

[ece310371-bib-0027] Brown, L. H. , Urban, E. K. , & Newman, K. (1982). The birds of Africa (Vol. I). Academic Press.

[ece310371-bib-0028] Buechley, E. R. , Oppel, S. , Efrat, R. , Phipps, W. L. , Carbonell Alanís, I. , Álvarez, E. , Andreotti, A. , Arkumarev, V. , Berger‐Tal, O. , Bermejo Bermejo, A. , Bounas, A. , Ceccolini, G. , Cenerini, A. , Dobrev, V. , Duriez, O. , García, J. , García Ripollés, C. , Galán, M. , Gil, A. , … Marra, P. P. (2021). Differential survival throughout the full annual cycle of a migratory bird presents a life‐history trade‐off. Journal of Animal Ecology, 90, 1228–1238. 10.1111/1365-2656.13449 33786863

[ece310371-bib-0029] Burnham, K. P. , & Anderson, D. R. (2002). Model selection and multimodel inference: A practical information‐theoretic approach (2nd ed.). Springer. 10.1007/b97636

[ece310371-bib-0030] Byholm, P. , Ranta, E. , Kaitala, V. , Lindén, H. , Saurola, P. , & Wikman, M. (2002). Resource availability and goshawk offspring sex ratio variation: A large‐scale ecological phenomenon. Journal of Animal Ecology, 71(6), 994–1001. 10.1046/j.1365-2656.2002.00663.x

[ece310371-bib-0031] Carranza, J. (2004). Sex allocation within broods: The intrabrood sharing‐out hypothesis. Behavioral Ecology, 15(2), 223–232. 10.1093/beheco/arh004

[ece310371-bib-0032] Carrete, M. , Donázar, J. A. , & Margalida, A. (2006). Density‐dependent productivity depression in Pyrenean bearded vultures: Implications for conservation. Ecological Applications, 16(5), 1674–1682. 17069362 10.1890/1051-0761(2006)016[1674:dpdipb]2.0.co;2

[ece310371-bib-0033] Carrete, M. , Donázar, J. A. , Margalida, A. , & Bertran, J. (2006). Linking ecology, behaviour and conservation: Does habitat saturation change the mating system of bearded vultures ? Biology Letters, 2, 624–627. 10.1098/rsbl.2006.0498 17148305 PMC1834000

[ece310371-bib-0034] Ceballos, O. , & Donázar, J. A. (1990). Roost‐tree characteristics, food habits and seasonal abundance of roosting Egyptian vultures in northern Spain. Journal of Raptor Research, 24(1–2), 19–25.

[ece310371-bib-0035] Clutton‐Brock, T. H. (1986). Sex ratio variation in birds. Ibis, 128(3), 317–329. 10.1111/j.1474-919X.1986.tb02682.x

[ece310371-bib-0036] Covas, R. (2016). Life‐history evolution in Island populations of birds. In R. M. Kliman (Ed.), Encyclopedia of evolutionary biology (Vol. 2, pp. 352–358). Oxford Academic Press. 10.1016/B978-0-12-800049-6.00099-8

[ece310371-bib-0037] Cramp, S. , & Simmons, K. E. L. (Eds.). (1980). Handbook of the birds of Europe, the Middle East and North Africa. The Birds of the Western Palearctic. Volume II. Hawks to bustards. Oxford University Press.

[ece310371-bib-0038] Curio, E. (1983). Why do young birds reproduce less well? Ibis, 125(3), 400–404. 10.1111/j.1474-919X.1983.tb03130.x

[ece310371-bib-0039] Daan, S. , Dijkstra, C. , & Weissing, F. J. (1996). An evolutionary explanation for seasonal trends in avian sex ratios. Behavioral Ecology, 7(4), 426–430. 10.1093/beheco/7.4.426

[ece310371-bib-0040] Davidovic, S. , Marinkovic, S. , Hribsek, I. , Patenkovic, A. , Stamenkovic‐Radak, M. , & Tanaskovic, M. (2022). Sex ratio and relatedness in the griffon vulture (*Gyps fulvus*) population of Serbia. PeerJ, 10, 1–21. 10.7717/peerj.14477 PMC9745909 36523455

[ece310371-bib-0041] Del Moral, J. C. , & Molina, B. (Eds.). (2018). El alimoche común en España, población reproductora en 2018 y método de censo. SEO/BirdLife.

[ece310371-bib-0042] Dijkstra, C. , Daan, S. , & Pen, I. (1998). Fledgling sex ratios in relation to brood size in size‐dimorphic altricial birds. Behavioral Ecology, 9(3), 287–296. 10.1093/beheco/9.3.287

[ece310371-bib-0043] Donald, P. F. (2007). Adult sex ratios in wild bird populations. Ibis, 149(4), 671–692. 10.1111/j.1474-919X.2007.00724.x

[ece310371-bib-0044] Donázar, J. A. (1993). In J. M. Reyero (Ed.), Los buitres ibéricos: biología y conservación (p. 256). Cornell University.

[ece310371-bib-0045] Donázar, J. A. , Barbosa, J. M. , García‐Alfonso, M. , van Overveld, T. , Gangoso, L. , & de la Riva, M. (2020). Too much is bad: Increasing numbers of livestock and conspecifics reduce body mass in an avian scavenger. Ecological Applications, 30, e02125. 10.1002/eap.2125 32167643

[ece310371-bib-0046] Donázar, J. A. , & Ceballos, O. (1988). Alimentación y tasas reproductoras del alimoche (*Neophron percnopterus*) en Navarra. Ardeola, 35(1), 3–14.

[ece310371-bib-0047] Donázar, J. A. , & Ceballos, O. (1989). Growth rates of nestling Egyptian vultures *Neophron percnopterus* in relation to brood size, hatching order and environmental factors. Ardea, 77(2), 217–226.

[ece310371-bib-0048] Donázar, J. A. , & Ceballos, O. (1990). Post‐fledging dependence period and development of flight and foraging behaviour in the Egyptian vulture *Neophron percnopterus* . Ardea, 78(3), 387–394.

[ece310371-bib-0049] Donázar, J. A. , Ceballos, O. , & Tella, J. L. (1994). Copulation behaviour in the Egyptian vulture *Neophron percnopterus* . Bird Study, 41(1), 37–41. 10.1080/00063659409477195

[ece310371-bib-0050] Donázar, J. A. , Cortés‐Avizanda, A. , Ceballos, O. , Arrondo, E. , Grande, J. M. , & Serrano, D. (2020). Epizootics and sanitary regulations drive long‐term changes in fledgling body condition of a threatened vulture. Ecological Indicators, 113, 1–7. 10.1016/j.ecolind.2020.106188

[ece310371-bib-0051] Donázar, J. A. , Gangoso, L. , Forero, M. G. , & Juste, J. (2005). Presence, richness and extinction of birds of prey in the Mediterranean and Macaronesian islands. Journal of Biogeography, 32(10), 1701–1713. 10.1111/j.1365-2699.2005.01294.x

[ece310371-bib-0052] Donázar, J. A. , Margalida, A. , & Campión, D. (Eds.). (2009). Vultures, feeding stations and sanitary legislation: A conflict and its consequences from the perspective of conservation biology (Munibe, 29). Sociedad de Ciencias Aranzadi.

[ece310371-bib-0053] Donázar, J. A. , Margalida, A. , Carrete, M. , & Sánchez‐Zapata, J. A. (2009). Too sanitary for vultures. Science, 326(5953), 664. 10.1126/science.326_664a 19900914

[ece310371-bib-0054] Donázar, J. A. , Palacios, C. J. , Gangoso, L. , Ceballos, O. , González, M. J. , & Hiraldo, F. (2002). Conservation status and limiting factors in the endangered population of Egyptian vulture (*Neophron percnopterus*) in the Canary Islands. Biological Conservation, 107, 89–97. 10.1016/S0006-3207(02)00049-6

[ece310371-bib-0055] Dzus, E. H. , Bortolotti, G. R. , & Gerrard, J. M. (1996). Does sex‐biased hatching order in bald eagles vary with food resources? Ecoscience, 3(3), 252–258. 10.1080/11956860.1996.11682339

[ece310371-bib-0056] Ellegren, H. , Gustafsson, L. , & Sheldon, B. C. (1996). Sex ratio adjustment in relation to paternal attractiveness in a wild bird population. Proceedings of the National Academy of Sciences of the United States of America, 93(21), 11723–11728. 10.1073/pnas.93.21.11723 8876204 PMC38125

[ece310371-bib-0057] Ferrer, M. , Bildstein, K. , Penteriani, V. , Casado, E. , & de Lucas, M. (2011). Why birds with deferred sexual maturity are sedentary on islands: A systematic review. PLoS One, 6(7), 1–7. 10.1371/journal.pone.0022056 PMC3139619 21811559

[ece310371-bib-0058] Ferrer, M. , & Donazar, J. A. (1996). Density‐dependent fecundity by habitat heterogeneity in an increasing population of Spanish imperial eagles. Ecology, 77(1), 69–74. 10.2307/2265655

[ece310371-bib-0059] Ferrer, M. , Newton, I. , & Pandolfi, M. (2009). Small populations and offspring sex‐ratio deviations in eagles. Conservation Biology, 23(4), 1017–1025. 10.1111/j.1523-1739.2009.01215.x 19627325

[ece310371-bib-0060] Ferrer, M. , Otalora, F. , & García‐Ruiz, J. M. (2004). Density‐dependent age of first reproduction as a buffer affecting persistence of small populations. Ecological Applications, 14(2), 616–624. 10.1890/02-5361

[ece310371-bib-0061] Fisher, R. A. (1930). The genetical theory of natural selection. Oxford University Press.

[ece310371-bib-0062] Forslund, P. , & Pärt, T. (1995). Age and reproduction in birds – Hypotheses and tests. Trends in Ecology & Evolution, 10(9), 374–378. 10.1016/S0169-5347(00)89141-7 21237076

[ece310371-bib-0063] Fridolfsson, A.‐K. , & Ellegren, H. (1999). A simple and universal method for molecular sexing of non‐ratite birds. Journal of Avian Biology, 30(1), 116–121. 10.1163/003925995X00233

[ece310371-bib-0064] Frumkin, R. (1989). Egg quality, nestling development and dispersal in the Sparrowhawk (*Accipiter nisus*). Journal of Raptor Research, 23(3), 123.

[ece310371-bib-0065] Gangoso, L. (2006). Insularidad y Conservación: el caso del alimoche (Neophron percnopterus) en Canarias. Universidad de Sevilla.

[ece310371-bib-0066] Gangoso, L. , Álvarez‐Lloret, P. , Rodríguez‐Navarro, A. A. B. , Mateo, R. , Hiraldo, F. , & Donázar, J. A. (2009). Long‐term effects of lead poisoning on bone mineralization in vultures exposed to ammunition sources. Environmental Pollution, 157(2), 569–574. 10.1016/j.envpol.2008.09.015 18995938

[ece310371-bib-0067] García‐Alfonso, M. , Van Overveld, T. , Gangoso, L. , Serrano, D. , & Donázar, J. A. (2020). Vultures and livestock: The where, when, and why of visits to farms. Animals, 10, 2127. 10.3390/ani10112127 33207713 PMC7698296

[ece310371-bib-0068] García‐Heras, M.‐S. , Cortés‐Avizanda, A. , & Donázar, J.‐A. (2013). Who are we feeding? Asymmetric individual use of surplus food resources in an insular population of the endangered Egyptian vulture *Neophron percnopterus* . PLoS One, 8(11), 1–7. 10.1371/journal.pone.0080523 PMC3823625 24244695

[ece310371-bib-0069] Gómez‐López, G. , Martínez, F. , Sanz‐Aguilar, A. , Carrete, M. , & Blanco, G. (2022). Nestling sex ratio is unaffected by individual and population traits in the griffon vulture. Current Zoology, 69(3), 227–235. 37351302 10.1093/cz/zoac046 PMC10284052

[ece310371-bib-0070] Gowaty, P. A. (1993). Differential dispersal, local resource competition, and sex ratio variation in birds. American Naturalist, 141(2), 263–280. 10.1086/285472 19426081

[ece310371-bib-0071] Grande, J. M. (2006). Factores Limitantes Antrópicos y Naturales de Poblaciones de Aves Carroñeras: El Caso del Alimoche (Neophron percnopterus) en el Valle del Ebro [Universidad de Sevilla] . http://digital.csic.es/handle/10261/64267

[ece310371-bib-0072] Grande, J. M. , Serrano, D. , Tavecchia, G. , Carrete, M. , Ceballos, O. , Díaz‐Delgado, R. , Tella, J. L. , & Donázar, J. A. (2009). Survival in a long‐lived territorial migrant: Effects of life‐history traits and ecological conditions in wintering and breeding areas. Oikos, 118, 580–590. 10.1111/j.1600-0706.2009.17218.x

[ece310371-bib-0073] Greenwood, P. J. (1980). Mating systems, philopatry and dispersal in birds and mammals. Animal Behaviour, 28(4), 1140–1162. 10.1016/S0003-3472(80)80103-5

[ece310371-bib-0074] Griffiths, R. , Daan, S. , & Dijkstra, C. (1996). Sex identification in birds using two CHD genes. Proceedings of the Royal Society B: Biological Sciences, 263(1374), 1251–1256. 10.1098/rspb.1996.0184 8858876

[ece310371-bib-0075] Griggio, M. , Hamerstrom, F. , Rosenfield, R. N. , & Tavecchia, G. (2002). Seasonal variation in sex ratio of fledgling American kestrels: A long term study. Wilson Bulletin, 114(4), 474–478. 10.1676/0043-5643(2002)114[0474:SVISRO]2.0.CO;2

[ece310371-bib-0076] Hartig, F. (2022). DHARMa: Residual diagnostics for Hierarchical (multi‐level/mixed) regression models (R Package) . https://cran.r‐project.org/web/packages/DHARMa/index.html

[ece310371-bib-0077] Hasselquist, D. , & Kempenaers, B. (2002). Parental care and adaptive brood sex ratio manipulation in birds. Philosophical Transactions of the Royal Society B: Biological Sciences, 357(1419), 363–372. 10.1098/rstb.2001.0924 PMC1692942 11958704

[ece310371-bib-0078] Heinsohn, R. , Legge, S. , & Barry, S. (1997). Extreme bias in sex allocation in *Eclectus* parrots. Proceedings of the Royal Society B: Biological Sciences, 264(1386), 1325–1329. 10.1098/rspb.1997.0183

[ece310371-bib-0079] Hörnfeldt, B. , Hipkiss, T. , Fridolfsson, A. K. , Eklund, U. , & Ellegren, H. (2000). Sex ratio and fledging success of supplementary‐fed Tengmalm's owl broods. Molecular Ecology, 9(2), 187–192. 10.1046/j.1365-294X.2000.00847.x 10672162

[ece310371-bib-0080] Iñigo, A. , & Atienza, J. C. (2007). Efectos del Reglamento 1774/2002 y las decisiones adoptadas por la Comisión Europea en 2003 y 2005 sobre las aves necrófagas en la península Ibérica y sus posibles soluciones . http://www.colectivoazalvaro.com/wp‐content/uploads/aa6017a271c3115c3b912adededea27e.pdf

[ece310371-bib-0081] Komdeur, J. , & Pen, I. (2002). Adaptive sex allocation in birds: The complexities of linking theory and practice. Philosophical Transactions of the Royal Society B: Biological Sciences, 357(1419), 373–380. 10.1098/rstb.2001.0927 PMC1692946 11958705

[ece310371-bib-0082] Kretzmann, M. B. , Capote, N. , Gautschi, B. , Godoy, J. A. , Donázar, J. A. , & Negro, J. J. (2003). Genetically distinct Island populations of the Egyptian vulture (*Neophron percnopterus*). Conservation Genetics, 4, 697–706.

[ece310371-bib-0083] Kumar, C. , Kaleka, A. S. , & Thind, S. K. (2020). Observations on breeding behaviour of a pair of endangered Egyptian vultures *Neophron percnopterus* (Linnaeus, 1758) over three breeding seasons in the plains of Punjab, India. Journal of Threatened Taxa, 12(9), 16013–16020. 10.11609/jott.4539.12.9.16013-16020

[ece310371-bib-0084] Lande, R. (1988). Genetics biological demography in conservation. Science, 241, 1455–1460. 3420403 10.1126/science.3420403

[ece310371-bib-0085] Legge, S. , Heinsohn, R. , Double, M. C. , Griffiths, R. , & Cockburn, A. (2001). Complex sex allocation in the laughing kookaburra. Behavioral Ecology, 12(5), 524–533. 10.1093/beheco/12.5.524

[ece310371-bib-0086] Lenth, R. (2016). Least‐squares means: The R package lsmeans. Journal of Statistical Software, 69(1), 1–33. 10.18637/jss.v069.i01

[ece310371-bib-0087] Leroux, A. , & Bretagnolle, V. (1996). Sex ratio variations in broods of Montagu's harriers *Circus pygargus* . Journal of Avian Biology, 27(1), 63–69. 10.2307/3676962

[ece310371-bib-0088] Lieury, N. , Gallardo, M. , Ponchon, C. , Besnard, A. , & Millon, A. (2015). Relative contribution of local demography and immigration in the recovery of a geographically‐isolated population of the endangered Egyptian vulture. Biological Conservation, 191, 349–356. 10.1016/j.biocon.2015.07.008

[ece310371-bib-0089] López‐Bao, J. V. , & Margalida, A. (2018). Slow transposition of European environmental policies. Nature Ecology and Evolution, 2(6), 914. 10.1038/s41559-018-0565-8 29735989

[ece310371-bib-0090] López‐López, P. , Gil, J. A. , & Alcántara, M. (2011). Morphometrics and sex determination in the endangered bearded vulture (*Gypaetus barbatus*). Journal of Raptor Research, 45(4), 361–366.

[ece310371-bib-0091] Losos, J. B. , & Ricklefs, R. E. (2009). Adaptation and diversification on islands. Nature, 457, 830–836. 10.1038/nature07893 19212401

[ece310371-bib-0092] Margalida, A. , Benítez, J. R. , Sánchez‐Zapata, J. A. , Ávila, E. , Arenas, R. , & Donázar, J. A. (2011). Long‐term relationship between diet breadth and breeding success in a declining population of Egyptian vultures *Neophron percnopterus* . Ibis, 154, 184–188. 10.1111/j.1474-919X.2011.01189.x

[ece310371-bib-0093] Mayr, E. (1939). The sex ratio in wild birds. The American Naturalist, 73(745), 156–179. 10.1086/280824

[ece310371-bib-0094] McDonald, P. G. , Olsen, P. D. , & Cockburn, A. (2005). Sex allocation and nestling survival in a dimorphic raptor: does size matter? Behavioral Ecology, 16(5), 922–930. 10.1093/beheco/ari071

[ece310371-bib-0095] Medina, F. M. (1999). Alimentación del alimoche, *Neophron percnopterus* (L.), en Fuerteventura, Islas Canarias (Aves, Accipitridae). Vieraea, 27, 77–86.

[ece310371-bib-0096] Mora, O. , Delgado, M. , & Penteriani, V. (2010). Secondary sex ratio in Eurasian eagle‐owls: Early‐breeding females produce more daughters. Journal of Raptor Research, 44(1), 62–65.

[ece310371-bib-0097] Morales‐Reyes, Z. , Pérez‐García, J. M. , Moleón, M. , Botella, F. , Carrete, M. , Donázar, J. A. , Cortés‐Avizanda, A. , Arrondo, E. , Moreno‐Opo, R. , Jiménez, J. , Margalida, A. , & Sánchez‐Zapata, J. A. (2017). Evaluation of the network of protection areas for the feeding of scavengers in Spain: From biodiversity conservation to greenhouse gas emission savings. Journal of Applied Ecology, 54, 1120–1129. 10.1111/1365-2664.12833

[ece310371-bib-0098] Morandini, V. , Muriel, R. , Newton, I. , & Ferrer, M. (2019). Skewed sex ratios in a newly established osprey population. Journal of Ornithology, 160(4), 1025–1033. 10.1007/s10336-019-01680-9

[ece310371-bib-0099] Morant, J. , López‐López, P. , & Zuberogoitia Arroyo, I. (2019). Parental investment asymmetries of a globally endangered scavenger: Unravelling the role of gender, weather conditions and stage of the nesting cycle. Bird Study, 66(3), 329–341. 10.1080/00063657.2019.1688251

[ece310371-bib-0100] Nager, R. G. , Monaghan, P. , Houston, D. C. , & Genovart, M. (2000). Parental condition, brood sex ratio and differential young survival: An experimental study in gulls (*Larus fuscus*). Behavioral Ecology and Sociobiology, 48(6), 452–457.

[ece310371-bib-0101] Navara, K. J. (Ed.) (2018). Potential mechanisms of sex ratio adjustment in birds. In Choosing sexes (pp. 99–121). Springer.

[ece310371-bib-0102] Negro, J. J. , Grande, J. M. , Tella, J. L. , Garrido, J. , Hornero, D. , Donázar, J. A. , Sanchez‐Zapata, J. A. , Benítez, J. R. , & Barcell, M. (2002). An unusual source of essential carotenoids. Nature, 416(6883), 807–808. 10.1038/416807a 11976670

[ece310371-bib-0103] Nisbet, I. C. , & Hatch, J. J. (1999). Consequences of a female‐biased sex‐ratio in a socially monogamous bird: Female‐female pairs in the roseate tern *Sterna dougallii* . Ibis, 141, 307–320.

[ece310371-bib-0104] Olsen, P. D. , & Cockburn, A. (1991). Female‐biased sex allocation in peregrine falcons and other raptors. Behavioral Ecology & Sociobiology, 28(6), 417–423. 10.1007/bf00164123

[ece310371-bib-0105] Oppel, S. , Arkumarev, V. , Bakari, S. , Dobrev, V. , Saravia, V. , Adefolu, S. , Aktay Sözüer, L. , Apeverga, P. T. , Arslan, Ş. , Barshep, Y. , Bino, T. , Bounas, A. , Çetin, T. , Dayyoub, M. , Dobrev, D. , Duro, K. , El‐Moghrabi, L. , ElSafoury, H. , Endris, A. , … Nikolov, S. C. (2021). Major threats to a migratory raptor vary geographically along the eastern Mediterranean flyway. Biological Conservation, 262(109277), 109277.

[ece310371-bib-0106] Oppel, S. , Saravia, V. , Bounas, A. , Arkumarev, V. , Kret, E. , Dobrev, V. , Dobrev, D. , Kordopatis, P. , Skartsi, T. , Velevski, M. , Petrovski, N. , Bino, T. , Topi, M. , Klisurov, I. , Stoychev, S. , & Nikolov, S. C. (2021). Population reinforcement and demographic changes needed to stabilise the population of a migratory vulture. Journal of Applied Ecology, 58, 2711–2721. 10.1111/1365-2664.13958

[ece310371-bib-0107] Payevsky, V. A. (2021). Sex ratio and sex‐specific survival in avian populations: A review. Biology Bulletin Reviews, 11(3), 317–327. 10.1134/S2079086421030099

[ece310371-bib-0108] Pike, T. W. , & Petrie, M. (2003). Potential mechanisms of avian sex manipulation. Biological Reviews of the Cambridge Philosophical Society, 78(4), 553–574. 10.1017/S1464793103006146 14700391

[ece310371-bib-0109] Redondo, T. , Romero, J. M. , Díaz‐Delgado, R. , & Nagy, J. (2019). Broodmate aggression and life history variation in accipitrid birds of prey. Ecology and Evolution, 9(16), 9185–9206. 10.1002/ece3.5466 31463015 PMC6706193

[ece310371-bib-0110] Riedstra, B. , Dijkstra, C. , & Daan, S. (1998). Daily energy expenditure of male and female marsh harrier nestlings. The Auk, 115(3), 635–641.

[ece310371-bib-0111] Ristow, D. , & Wink, M. (2004). Seasonal variation in sex ratio of nestling Eleonora's falcons. Journal of Raptor Research, 38(4), 320–325.

[ece310371-bib-0112] Rosenfield, R. N. , Bielefeldt, J. , & Vos, S. M. (1996). Skewed sex ratios in Cooper's hawk offspring. Auk, 113(4), 957–960. 10.2307/4088881

[ece310371-bib-0113] Rosenfield, R. N. , Stout, W. E. , Giovanni, M. D. , Levine, N. H. , Cava, J. A. , Hardin, M. G. , & Haynes, T. G. (2015). Does breeding population trajectory and age of nesting females influence disparate nestling sex ratios in two populations of Cooper's hawks? Ecology and Evolution, 5(18), 4037–4048. 10.1002/ece3.1674 26445658 PMC4588665

[ece310371-bib-0114] RStudioTeam . (2021). RStudio: Integrated development for R. RStudio, PBC. http://www.rstudio.com/

[ece310371-bib-0115] Rutz, C. (2012). Brood sex ratio varies with diet composition in a generalist raptor. Biological Journal of the Linnean Society, 105(4), 937–951. 10.1111/j.1095-8312.2011.01818.x

[ece310371-bib-0116] Sanz‐Aguilar, A. , Béchet, A. , Germain, C. , Johnson, A. R. , & Pradel, R. (2012). To leave or not to leave: Survival trade‐offs between different migratory strategies in the greater flamingo. Journal of Animal Ecology, 81(6), 1171–1182. 10.1111/j.1365-2656.2012.01997.x 22612528

[ece310371-bib-0117] Sanz‐Aguilar, A. , Cortés‐Avizanda, A. , Serrano, D. , Blanco, G. , Ceballos, O. , Grande, J. M. , Tella, J. L. , & Donázar, J. A. (2017). Sex‐ and age‐dependent patterns of survival and breeding success in a long‐lived endangered avian scavenger. Scientific Reports, 7, 40204. 10.1038/srep40204 28074860 PMC5225485

[ece310371-bib-0118] Sanz‐Aguilar, A. , De Pablo, F. , & Donázar, J. A. (2015). Age‐dependent survival of Island vs. mainland populations of two avian scavengers: Delving into migration costs. Oecologia, 179(2), 405–414. 10.1007/s00442-015-3355-x 26013875

[ece310371-bib-0119] Sanz‐Aguilar, A. , Sánchez‐Zapata, J. A. , Carrete, M. , Ramón, J. , Ávila, E. , Arenas, R. , & Donázar, J. A. (2015). Action on multiple fronts, illegal poisoning and wind farm planning, is required to reverse the decline of the Egyptian vulture in southern Spain. Biological Conservation, 187, 10–18. 10.1016/j.biocon.2015.03.029

[ece310371-bib-0120] Sergio, F. , Tanferna, A. , De Stephanis, R. , López Jiménez, L. , Blas, J. , Tavecchia, G. , Preatoni, D. , & Hiraldo, F. (2014). Individual improvements and selective mortality shape lifelong migratory performance. Nature, 515(7527), 410–413. 10.1038/nature13696 25252973

[ece310371-bib-0121] Serrano, D. , Carrete, M. , & Tella, J. L. (2008). Describing dispersal under habitat constraints: A randomization approach in lesser kestrels. Basic and Applied Ecology, 9(6), 771–778. 10.1016/j.baae.2007.08.013

[ece310371-bib-0122] Serrano, D. , Cortés‐Avizanda, A. , Zuberogoitia, I. , Blanco, G. , Benítez, J. R. , Ponchon, C. , Grande, J. M. , Ceballos, O. , Morant, J. , Arrondo, E. , Zabala, J. , Montelío, E. , Ávila, E. , González, J. L. , Arroyo, B. , Frías, Ó. , Kobierzycki, E. , Arenas, R. , Tella, J. L. , & Donázar, J. A. (2021). Phenotypic and environmental correlates of natal dispersal in a long‐lived territorial vulture. Scientific Reports, 11(5424), 1–14. 10.1038/s41598-021-84811-8 33686130 PMC7970891

[ece310371-bib-0123] Sheldon, B. C. , Kruuk, L. E. B. , & Alberts, S. C. (2022). The expanding value of long‐term studies of individuals in the wild. Nature Ecology and Evolution., 6, 1799–1801. 10.1038/s41559-022-01940-7 36348080

[ece310371-bib-0124] Simmons, R. (1988). Offspring quality and the evolution of cainism. Ibis, 130(3), 339–357. 10.1111/j.1474-919X.1988.tb08809.x

[ece310371-bib-0125] Slagsvold, T. (1990). Fisher's sex ratio theory may explain hatching patterns in birds. Evolution, 44(4), 1009–1017. 10.1111/j.1558-5646.1990.tb03821.x 28569020

[ece310371-bib-0126] Slagsvold, T. , Roskaft, E. , & Engen, S. (1986). Sex ratio, differential cost of rearing young, and differential mortality between the sexes during the period of parental care: Fisher's theory applied to birds. Ornis Scandinavica, 17(2), 117–125. 10.2307/3676860

[ece310371-bib-0127] Smallwood, P. D. , & Smallwood, J. A. (1998). Seasonal shifts in sex ratios of fledgling American kestrels (*Falco sparverius paulus*): The early bird hypothesis. Evolutionary Ecology, 12(7), 839–853. 10.1023/A:1006598600532

[ece310371-bib-0128] Stoffel, M. A. , Nakagawa, S. , & Schielzeth, H. (2017). rptR: Repeatability estimation and variance decomposition by generalized linear mixed‐effects models. Methods in Ecology and Evolution, 8(11), 1639–1644. 10.1111/2041-210X.12797

[ece310371-bib-0129] Szász, E. , Kiss, D. , & Rosivall, B. (2012). Sex ratio adjustment in birds. Ornis Hungarica, 20(1), 26–36. 10.2478/orhu-2013-0002

[ece310371-bib-0130] Székely, T. , Thomas, G. H. , & Cuthill, I. C. (2006). Sexual conflict, ecology, and breeding systems in shorebirds. Bioscience, 56(10), 801–808. 10.1641/0006-3568(2006)56[801:SCEABS]2.0.CO;2

[ece310371-bib-0131] Tella, J. L. , Donazar, J. A. , Negro, J. J. , & Hiraldo, F. (1996). Seasonal and interannual variations in the sex‐ratio of lesser kestrel *Falco naumanni* broods. Ibis, 138(2), 342–345. 10.1111/j.1474-919x.1996.tb04350.x

[ece310371-bib-0132] Tella, J. L. (1993). Polyandrous trios in a population of Egyptian vultures (*Neophron percnopterus*). Journal of Raptor Research, 27(2), 119–120.

[ece310371-bib-0133] Trivers, R. L. , & Willard, D. E. (1973). Natural selection of parental ability to vary the sex ratio of offspring. Science, 179(4068), 90–92. 10.1126/science.179.4068.90 4682135

[ece310371-bib-0134] Tschumi, M. , Humbel, J. , Erbes, J. , Fattebert, J. , Fischer, J. , Fritz, G. , Geiger, B. , Van Harxen, R. , Hoos, B. , Hurst, J. , Jacobsen, L. B. , Keil, H. , Kneule, W. , Michel, V. T. , Michels, H. , Möbius, L. , Perrig, M. , Rößler, P. , Schneider, D. , … Grüebler, M. U. (2019). Parental sex allocation and sex‐specific survival drive offspring sex ratio bias in little owls. Behavioral Ecology & Sociobiology, 73(85), 1–10.

[ece310371-bib-0135] Uller, T. (2006). Sex‐specific sibling interactions and offspring fitness in vertebrates: Patterns and implications for maternal sex ratios. Biological Reviews, 81(2), 207–217. 16677432 10.1017/S1464793105006962

[ece310371-bib-0136] Van Overveld, T. , Blanco, G. , Moleón, M. , Margalida, A. , Sánchez‐Zapata, J. A. , De la Riva, M. , & Donázar, J. A. (2020). Integrating vulture social behavior into conservation practice. The Condor, 122, 1–20. 10.1093/condor/duaa035

[ece310371-bib-0137] Van Overveld, T. , García‐Alfonso, M. , Dingemanse, N. J. , Bouten, W. , Gangoso, L. , De Riva, M. , Serrano, D. , & Donázar, J. A. (2018). Food predictability and social status drive individual resource specializations in a territorial vulture. Scientific Reports, 8, 1–14. 10.1038/s41598-018-33564-y 30310140 PMC6181911

[ece310371-bib-0138] Venables, J. C. , & Brooke, M. D. L. (2015). The comparative effects of small geographic range and population decline on the adult sex ratio of threatened bird species. Bird Conservation International, 25, 182–191. 10.1017/S0959270914000161

[ece310371-bib-0139] Villegas, A. , Sánchez‐Guzmán, J. M. , Costillo, E. , Corbacho, C. , & Morán, R. (2004). Productivity and fledgling sex ratio in a cinereous vulture (*Aegypius monachus*) population in Spain. Journal of Raptor Research, 38(4), 361–366.

[ece310371-bib-0140] Warkentin, I. G. , Brooks, D. W. , Lieske, D. J. , Espie, R. H. M. , & James, P. C. (2022). Brood sex ratios in Merlins reflect characteristics of the associated breeding male and population density. Ibis, 165, 533–545. 10.1111/ibi.13159

[ece310371-bib-0141] West, S. , Reece, S. , & Sheldon, B. (2002). Sex ratios. Heredity, 88, 117–124. 10.1038/sj/hdy/6800018 11932770

[ece310371-bib-0142] Whittaker, R. J. , & Fernández‐Palacios, J. M. (2007). Island biogeography: Ecology, evolution, and conservation. Oxford University Press.

[ece310371-bib-0143] Wiebe, K. L. , & Bortolotti, G. R. (1992). Facultative sex ratio manipulation in American kestrels. Behavioral Ecology and Sociobiology, 30, 379–386.

[ece310371-bib-0144] Xirouchakis, S. M. , Botsidou, P. , Baxevani, K. , Andreou, G. , & Tsaparis, D. (2022). Brood sex ratio variation in a colonial raptor, the Eleonora's falcon, *Falco eleonorae* . Animal Behaviour, 1–14, 93–106. 10.1016/j.anbehav.2022.11.001

[ece310371-bib-0145] Yordanov, E. , Dobrev, V. , Arkumarev, V. , Dobrev, D. , & Nicolov, S. C. (2021). Identifying hatchlings mortality in the Egyptian vulture (Neophron percnopterus) through the means of trail cameras. Technical report under action D1 of the LIFE project “Egyptian Vulture New LIFE” (LIFE16 NAT/BG/000874) .

