# Supplementary material for: Insularity determines nestling sex ratio variation in Egyptian vulture populations
Source: Ecol Evol. 2023 Jul 29;13(8):e10371. doi: 10.1002/ece3.10371 (PMC10385291; doi:10.1002/ece3.10371)
Supplement: Supplementary file 1 — Data S1 [file ECE3-13-e10371-s001.docx]

**SUPPORTING INFORMATION**

**Insularity determines nestling sex ratio variation in Egyptian vulture populations**

Guillermo Gómez-López, Ana Sanz-Aguilar, Martina Carrete, Eneko Arrondo, José Ramón Benítez, Olga Ceballos, Ainara Cortés-Avizanda, Félix de Pablo, José Antonio Donázar, Óscar Frías, Laura Gangoso, Marina García-Alfonso, José Luis González, Juan Manuel Grande, David Serrano, José Luis Tella, Guillermo Blanco

**1. Sex ratio analyses**


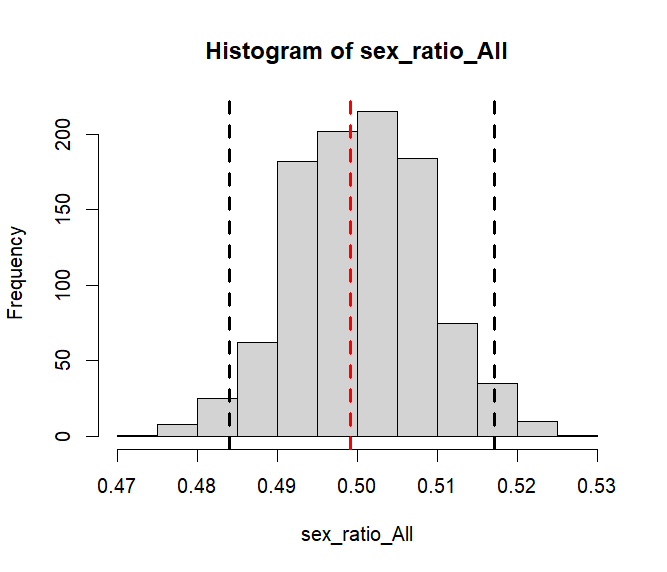

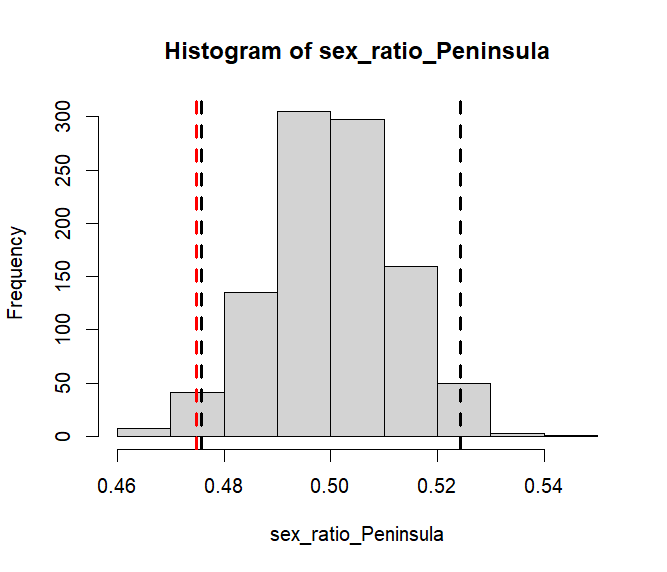

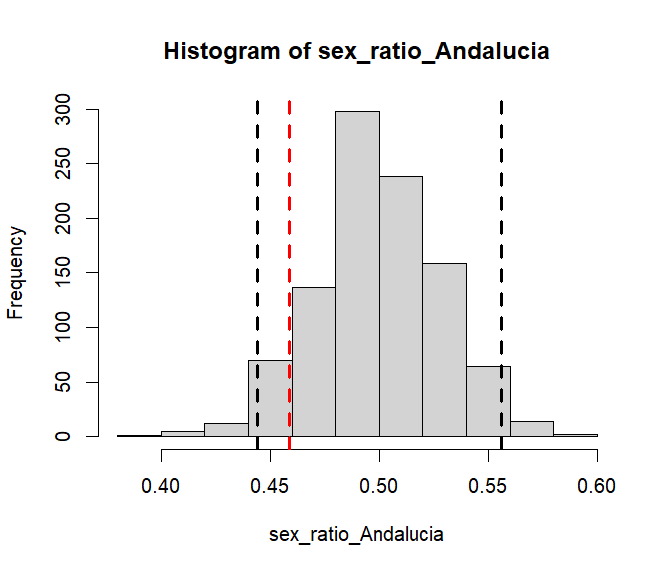

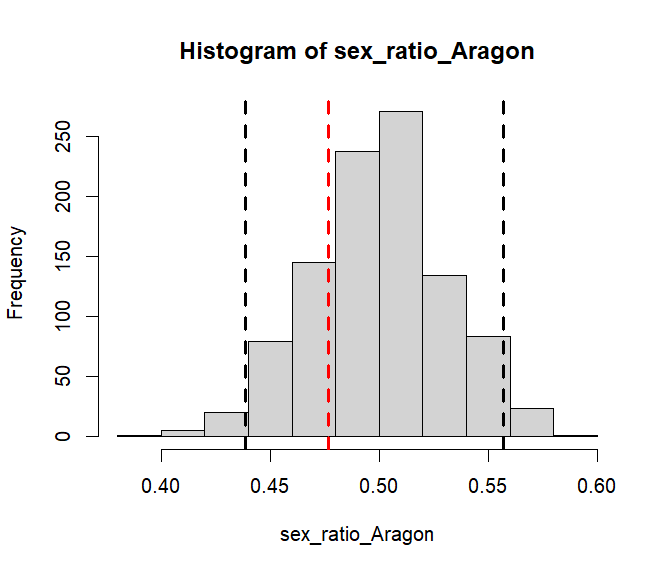

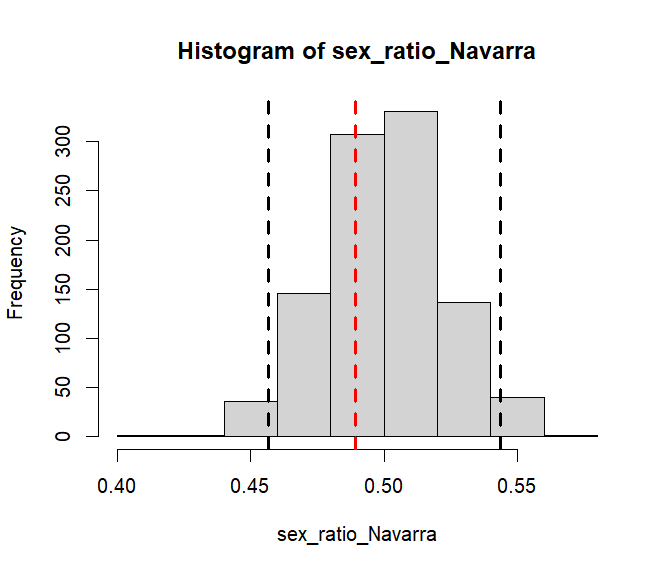

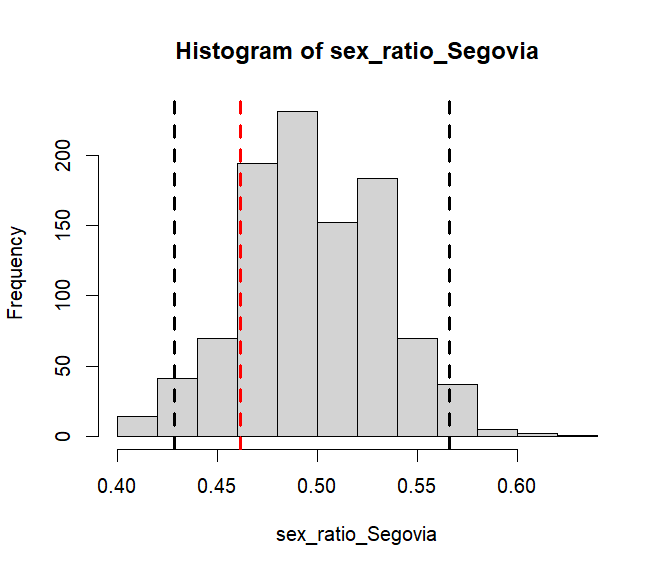

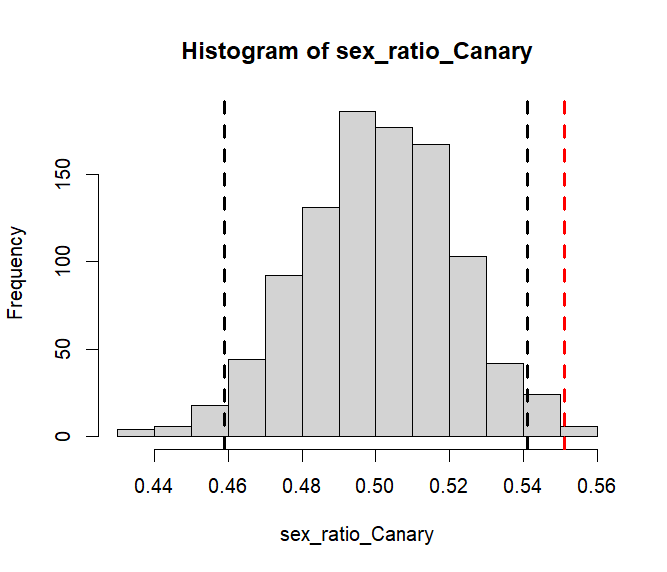

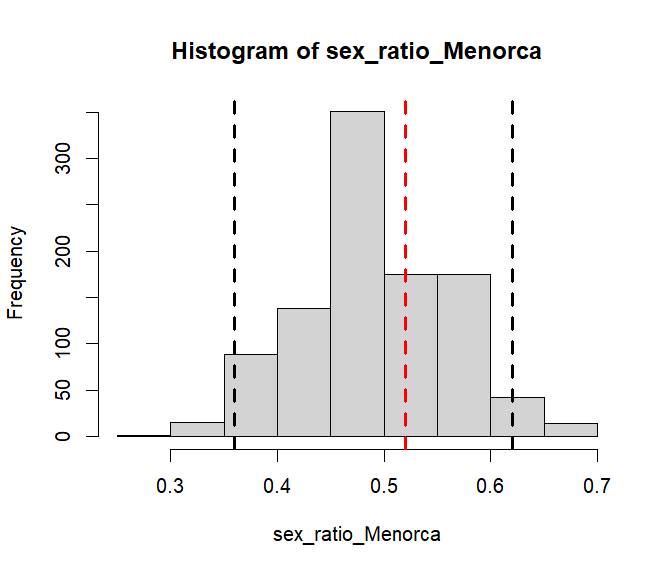


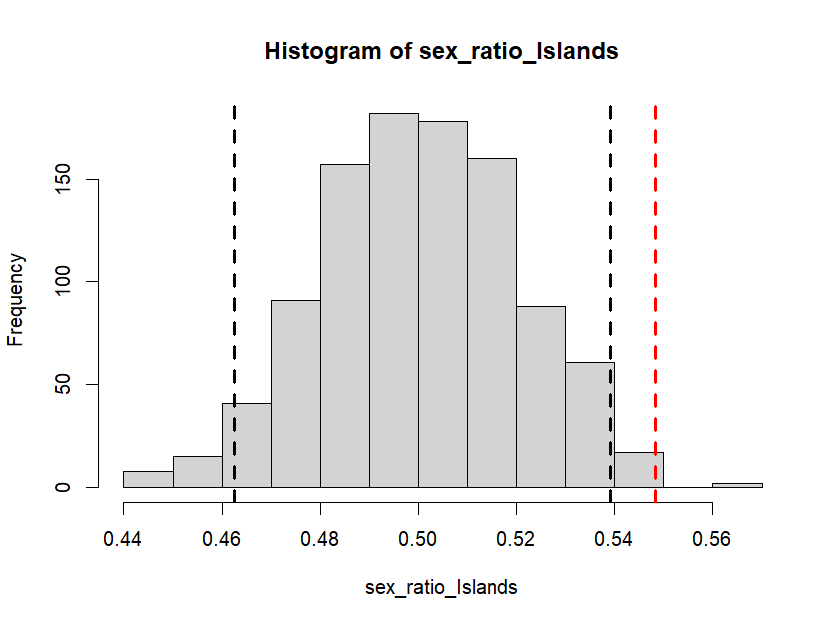


Figure S1. Observed (red line) and expected (black lines; 95% CI) offspring sex ratios under the assumption of a balanced sex ratio (1:1) considering region-specific sample sizes. For each breeding region, we ran 1,000 Monte Carlo simulations by randomly picking a number of nestlings equal to its sample size from a theoretical set of 3,000 nestlings with a balanced sex ratio (1:1) and calculating the resulting sex ratio.

Table S1. Output obtained to assess the effects of the year for each region (sex ~ year).

| Variable | Estimate | SE | 2.5% CI | 97.5% CI |
| --- | --- | --- | --- | --- |
| (i) *all nestlings* |  |  |  |  |
| Intercept | -0.03 | 0.23 | -0.49 | 0.43 |
| 1995 | -0.03 | 0.41 | -0.84 | 0.78 |
| 1996 | -1.01 | 0.53 | -2.05 | 0.02 |
| 1997 | -0.15 | 0.49 | -1.11 | 0.80 |
| 1998 | 0.41 | 0.41 | -0.39 | 1.21 |
| 1999 | 0.26 | 0.34 | -0.40 | 0.93 |
| 2000 | -0.10 | 0.30 | -0.68 | 0.48 |
| 2001 | -0.13 | 0.32 | -0.76 | 0.49 |
| 2002 | -0.38 | 0.30 | -0.96 | 0.21 |
| 2003 | -0.11 | 0.32 | -0.74 | 0.51 |
| 2004 | 0.28 | 0.34 | -0.40 | 0.95 |
| 2005 | -0.14 | 0.40 | -0.93 | 0.66 |
| 2006 | -0.15 | 0.38 | -0.91 | 0.60 |
| 2007 | -0.79 | 0.43 | -1.64 | 0.05 |
| 2008 | 0.41 | 0.43 | -0.43 | 1.25 |
| 2009 | -0.18 | 0.44 | -1.04 | 0.68 |
| 2010 | 0.52 | 0.37 | -0.22 | 1.25 |
| 2011 | -0.27 | 0.36 | -0.98 | 0.44 |
| 2012 | 0.17 | 0.39 | -0.59 | 0.94 |
| 2013 | 0.23 | 0.35 | -0.46 | 0.91 |
| 2015 | -0.00 | 0.34 | -0.68 | 0.67 |
| 2016 | 0.13 | 0.32 | -0.51 | 0.76 |
| 2017 | 0.27 | 0.34 | -0.40 | 0.94 |
| 2018 | 0.10 | 0.32 | -0.53 | 0.72 |
| 2019 | 0.09 | 0.35 | -0.59 | 0.77 |
| 2020 | -0.06 | 0.32 | -0.68 | 0.56 |
| 2021 | 0.61 | 0.32 | -0.01 | 1.23 |
| (ii) *peninsular Spain* |  |  |  |  |
| Intercept | -0.00 | 0.38 | -0.74 | 0.74 |
| 1995 | -0.06 | 0.51 | -1.05 | 0.94 |
| 1996 | -1.04 | 0.61 | -2.23 | 0.15 |
| 1997 | -0.18 | 0.57 | -1.30 | 0.94 |
| 1998 | 0.59 | 0.55 | -0.48 | 1.66 |
| 1999 | 0.15 | 0.47 | -0.77 | 1.07 |
| 2000 | -0.22 | 0.42 | -1.06 | 0.61 |
| 2001 | -0.34 | 0.45 | -1.23 | 0.55 |
| 2002 | -0.46 | 0.43 | -1.31 | 0.39 |
| 2003 | -0.18 | 0.47 | -1.09 | 0.73 |
| 2004 | 0.30 | 0.48 | -0.64 | 1.24 |
| 2005 | -0.69 | 0.60 | -1.86 | 0.48 |
| 2006 | -0.69 | 0.56 | -1.78 | 0.40 |
| 2007 | -0.62 | 0.60 | -1.80 | 0.56 |
| 2008 | 0.12 | 0.62 | -1.09 | 1.32 |
| 2009 | -0.29 | 0.60 | -1.58 | 1.00 |
| 2010 | 0.64 | 0.56 | -0.46 | 1.73 |
| 2011 | -0.53 | 0.52 | -1.54 | 0.48 |
| 2013 | 0.26 | 0.50 | -0.72 | 1.23 |
| 2014 | -0.10 | 0.49 | -1.07 | 0.87 |
| 2015 | -0.43 | 0.50 | -1.41 | 0.56 |
| 2016 | 0.39 | 0.49 | -0.58 | 1.35 |
| 2017 | -0.10 | 0.49 | -1.07 | 0.87 |
| 2018 | 0.00 | 0.47 | -0.93 | 0.93 |
| 2019 | -0.27 | 0.50 | -1.26 | 0.71 |
| 2020 | 0.07 | 0.46 | -0.84 | 0.98 |
| 2021 | 0.53 | 0.47 | -0.39 | 1.45 |
| (iii) *Canary Islands* |  |  |  |  |
| Intercept | -0.00 | 0.82 | -1.60 | 1.60 |
| 1999 | 1.25 | 1.11 | -0.99 | 3.50 |
| 2000 | 0.85 | 1.07 | -1.25 | 2.94 |
| 2001 | 0.00 | 0.98 | -1.91 | 1.91 |
| 2002 | 0.13 | 0.97 | -1.76 | 2.03 |
| 2003 | -0.61 | 0.96 | -2.49 | 1.28 |
| 2004 | 0.12 | 0.95 | -1.74 | 1.98 |
| 2005 | 0.51 | 0.97 | -1.38 | 2.40 |
| 2006 | 0.61 | 0.96 | -1.28 | 2.49 |
| 2007 | -1.10 | 1.00 | -3.06 | 0.86 |
| 2008 | 0.69 | 0.98 | -1.23 | 2.62 |
| 2009 | -0.13 | 0.97 | -2.03 | 1.76 |
| 2010 | 0.34 | 0.92 | -1.46 | 2.13 |
| 2011 | 0.11 | 0.94 | -1.73 | 1.94 |
| 2012 | 0.47 | 1.00 | -1.48 | 2.42 |
| 2013 | 0.10 | 0.93 | -1.72 | 1.91 |
| 2014 | 0.06 | 0.89 | -1.68 | 1.80 |
| 2015 | 0.58 | 0.92 | -1.22 | 2.37 |
| 2016 | -0.21 | 0.88 | -1.93 | 1.51 |
| 2017 | 0.81 | 0.92 | -0.99 | 2.61 |
| 2018 | 0.16 | 0.88 | -1.56 | 1.89 |
| 2019 | 0.63 | 0.93 | -1.19 | 2.44 |
| 2020 | -0.38 | 0.89 | -2.13 | 1.37 |
| 2021 | 0.66 | 0.88 | -1.07 | 2.38 |
| (iv) *Balearic Islands* |  |  |  |  |
| Intercept | -0.00 | 0.82 | -1.60 | 1.60 |
| 1998 | -0.69 | 1.47 | -3.58 | 2.19 |
| 1999 | -0.29 | 1.12 | -2.48 | 1.90 |
| 2001 | 1.10 | 1.16 | -1.16 | 3.36 |
| 2002 | -0.69 | 1.02 | -2.69 | 1.31 |
| 2003 | 0.59 | 0.99 | -1.35 | 2.53 |

Table S2. Models evaluating the effects of the age of the breeding males (age breeding male) and the age of the breeding females (age breeding female), using their raw age (see Materials and Methods for details), on the probability of a nestling Egyptian Vulture being a male in (A) peninsular Spain (*n* = 84) and (B) the Canary Islands (*n* = 237). Estimates, standard errors (SE), and 95% confidence intervals (CI) were obtained after model averaging. All models were run including male identity or female identity as random terms. The null model was included in our set of models. df: degrees of freedom; AICc: Akaike information criterion corrected for small sample sizes; ΔAICc: difference between the AICc of model *i* and that of the best model (i.e., the model with the lowest AICc); w: Akaike weight.

| Model selection | | | | |
| --- | --- | --- | --- | --- |
| Model | df | AICc | ΔAICc | w |
| (A) peninsular Spain |  |  |  |  |
| age breeding male | 3 | 47.94 | 0.00 | 0.57 |
| null | 2 | 48.54 | 0.59 | 0.43 |
| null | 2 | 85.17 | 0.00 | 0.66 |
| age breeding female | 3 | 86.54 | 1.36 | 0.34 |
| (B) Canary Islands |  |  |  |  |
| null | 2 | 239.84 | 0.00 | 0.73 |
| age breeding male | 3 | 241.82 | 1.98 | 0.27 |
| null | 2 | 180.68 | 0.00 | 0.64 |
| age breeding female | 3 | 181.83 | 1.15 | 0.36 |
| Model averaging | | | | |
| Variable | Estimate | SE | 2.5% CI | 97.5% CI |
| (A) |  |  |  |  |
| Intercept | 1.93 | 1.61 | -1.29 | 5.15 |
| age breeding male | -0.25 | 0.15 | -0.56 | 0.06 |
| Intercept | -0.55 | 0.78 | -2.09 | 1.00 |
| age breeding female | 0.07 | 0.08 | -0.08 | 0.23 |
| (B) |  |  |  |  |
| Intercept | 0.26 | 0.33 | -0.40 | 0.91 |
| age breeding male | 0.01 | 0.05 | -0.08 | 0.11 |
| Intercept | 0.46 | 0.46 | -0.45 | 1.37 |
| age breeding female | -0.05 | 0.05 | -0.15 | 0.05 |

Table S3. Models evaluating the effects of the age of the breeding males (age breeding male) and the age of the breeding females (age breeding female), using categorical ages (i) Badia-Boher et al. 2019 and (ii) Sanz-Aguilar et al. 2017 (see Materials and Methods for details), on the probability of a nestling Egyptian Vulture being a male in (A) peninsular Spain (*n* = 84) and (B) the Canary Islands (*n* = 237). Estimates, standard errors (SE), and 95% confidence intervals (CI) were obtained after model averaging. All models were run including male identity or female identity as random terms. The null model was included in our set of models. df: degrees of freedom; AICc: Akaike information criterion corrected for small sample sizes; ΔAICc: difference between the AICc of model *i* and that of the best model (i.e., the model with the lowest AICc); w: Akaike weight.

| Model selection | | | | |
| --- | --- | --- | --- | --- |
| Model | df | AICc | ΔAICc | w |
| (i) categorical age (Badia-Boher et al. 2019) |  |  |  |  |
| (A) *peninsular Spain* |  |  |  |  |
| null | 2 | 48.54 | 0.00 | 0.75 |
| age breeding male | 3 | 50.76 | 2.22 | 0.25 |
| null | 2 | 85.17 | 0.00 | 0.84 |
| age breeding female | 4 | 88.50 | 3.32 | 0.16 |
| (B) *Canary Islands* |  |  |  |  |
| null | 2 | 239.84 | 0.00 | 0.88 |
| age breeding male | 4 | 243.76 | 3.92 | 0.12 |
| null | 2 | 180.68 | 0.00 | 0.59 |
| age breeding female | 4 | 181.44 | 0.76 | 0.41 |
| (ii) categorical age (Sanz-Aguilar et al. 2017) |  |  |  |  |
| (A) |  |  |  |  |
| null | 2 | 48.54 | 0.00 | 0.61 |
| age breeding male | 3 | 49.41 | 0.87 | 0.39 |
| null | 2 | 85.17 | 0.00 | 0.68 |
| age breeding female | 3 | 86.73 | 1.55 | 0.32 |
| (B) |  |  |  |  |
| null | 2 | 239.84 | 0.00 | 0.74 |
| age breeding male | 3 | 241.90 | 2.05 | 0.26 |
| null | 2 | 180.68 | 0.00 | 0.68 |
| age breeding female | 3 | 182.21 | 1.53 | 0.32 |
| Model averaging | | | | |
| Variable | Estimate | SE | 2.5% CI | 97.5% CI |
| (i) categorical age (Badia-Boher et al. 2019) |  |  |  |  |
| (B) |  |  |  |  |
| Intercept | 0.33 | 0.20 | -0.07 | 0.72 |
| age breeding female (old) | -0.88 | 0.60 | -2.08 | 0.31 |
| age breeding female (subadult) | -0.92 | 0.76 | -2.42 | 0.57 |
| (i) categorical age (Sanz-Aguilar et al. 2017) |  |  |  |  |
| (A) |  |  |  |  |
| Intercept | 0.50 | 0.41 | -0.33 | 1.33 |
| age breeding male (young adult) | 1.05 | 0.89 | -0.77 | 2.87 |
| Intercept | -0.22 | 0.27 | -0.75 | 0.31 |
| age breeding female (young adult) | -0.92 | 1.19 | -3.29 | 1.46 |
| (B) |  |  |  |  |
| Intercept | 0.24 | 0.19 | -0.13 | 0.61 |
| age breeding female (young adult) | 0.33 | 0.45 | -0.55 | 1.22 |

Table S4. Models assessing the effects of hatching date (hatching date), region (region), insularity (insularity), food availability (mad-cows), brood size (brood size) and hatching order (order) on the probability of a nestling Egyptian Vulture being a male (*n* = 1,611). All models were run including year and territory as random terms. The null model was included in our set of models. df: degrees of freedom; AICc: Akaike information criterion corrected for small sample sizes; ΔAICc: difference between the AICc of model *i* and that of the best model (i.e., the model with the lowest AICc); w: Akaike weight. Nestlings from the Balearic Islands were excluded from this analysis since data on hatching date and order were unavailable.

| Model | df | AICc | ΔAICc | w |
| --- | --- | --- | --- | --- |
| insularity + brood size | 5 | 2230.89 | 0.00 | 0.19 |
| insularity | 4 | 2232.42 | 1.53 | 0.09 |
| insularity + mad-cows | 6 | 2232.66 | 1.76 | 0.08 |
| insularity + mad-cows + brood size + hatching date | 8 | 2232.67 | 1.78 | 0.08 |
| insularity + brood size + hatching date | 6 | 2232.73 | 1.84 | 0.08 |
| insularity + order | 6 | 2232.90 | 2.01 | 0.07 |
| insularity + hatching date | 5 | 2234.07 | 3.18 | 0.04 |
| mad-cows + brrod size + hatching date | 7 | 2234.09 | 3.20 | 0.04 |
| insularity + mad-cows + hatching date | 7 | 2234.21 | 3.32 | 0.04 |
| mad-cows + hatching date | 6 | 2234.24 | 3.35 | 0.04 |
| hatching date | 4 | 2234.50 | 3.61 | 0.03 |
| brood size + hatching date | 5 | 2234.66 | 3.77 | 0.03 |
| insularity + mad-cows + order + hatching date | 9 | 2234.67 | 3.78 | 0.03 |
| insularity + order + hatching date | 7 | 2234.72 | 3.83 | 0.03 |
| mad-cows + brood size + region | 10 | 2235.59 | 4.70 | 0.02 |
| mad-cows + order + hatching date | 8 | 2235.98 | 5.09 | 0.01 |
| brood size + region | 8 | 2236.21 | 5.32 | 0.01 |
| order + hatching date | 6 | 2236.54 | 5.65 | 0.01 |
| mad-cows | 5 | 2236.64 | 5.75 | 0.01 |
| mad-cows + region | 9 | 2236.92 | 6.03 | 0.01 |
| mad-cows + brood size | 6 | 2236.98 | 6.09 | 0.01 |
| null | 3 | 2237.09 | 6.20 | 0.01 |
| mad-cows + brood size + hatching date + region | 11 | 2237.34 | 6.45 | 0.01 |
| region | 7 | 2237.42 | 6.53 | 0.01 |
| mad-cows + order + region | 11 | 2237.61 | 6.72 | 0.01 |
| brood size | 4 | 2237.76 | 6.87 | 0.01 |
| brood size + hatching date + region | 9 | 2238.02 | 7.13 | 0.01 |
| order + region | 9 | 2238.22 | 7.33 | 0.00 |
| mad-cows + hatching date + region | 10 | 2238.42 | 7.52 | 0.00 |
| mad-cows + order | 7 | 2238.99 | 8.09 | 0.00 |
| hatching date + region | 8 | 2239.00 | 8.11 | 0.00 |
| mad-cows + order + hatching date + region | 12 | 2239.34 | 8.45 | 0.00 |
| order | 5 | 2239.76 | 8.87 | 0.00 |
| order + hatching date + region | 10 | 2240.02 | 9.13 | 0.00 |

Table S5. Estimates, SE and p-values of the different factors included in alternative models (ΔAICc < 2) in Table S4. In bold, significant effects (p<0.05).

| **Model** | **Variable** | **Estimate** | **SE** | **p-value** |
| --- | --- | --- | --- | --- |
| **insularity** + brood size | insularity (island) | 0.35 | 0.12 | **0.0023** |
|  | brood size (2) | 0.20 | 0.11 | 0.0605 |
| **insularity** | insularity (island) | 0.29 | 0.11 | **0.0078** |
| **insularity** + **mad-cows** | insularity (island) | 0.28 | 0.11 | **0.0123** |
|  | mad-cows (post) | 0.25 | 0.12 | **0.0405** |
|  | mad-cows (pre) | 0.11 | 0.14 | 0.4301 |
| insularity + **mad-cows** + brood size + hatching date | insularity (island) | 0.29 | 0.15 | 0.0602 |
|  | mad-cows (post) | 0.25 | 0.12 | **0.0331** |
|  | mad-cows (pre) | 0.10 | 0.14 | 0.4662 |
|  | brood size (2) | 0.20 | 0.11 | 0.0600 |
|  | hatching date | -0.03 | 0.07 | 0.6220 |
| **insularity** + brood size + hatching date | insularity (island) | 0.31 | 0.15 | **0.0439** |
|  | brood size (2) | 0.19 | 0.11 | 0.0675 |
|  | hatching date | -0.03 | 0.07 | 0.6731 |

Table S6. Models assessing the effects of hatching date (hatching date), region (region), food availability (mad-cows), brood size (brood size) and hatching order (order) on the probability of a nestling Egyptian Vulture being a male in peninsular Spain (*n* = 1,112). All models were run including year and territory as random terms. The null model was included in our set of models. df: degrees of freedom; AICc: Akaike information criterion corrected for small sample sizes; ΔAICc: difference between the AICc of model *i* and that of the best model (i.e., the model with the lowest AICc); w: Akaike weight.

| Model | df | AICc | ΔAICc | w |
| --- | --- | --- | --- | --- |
| null | 3 | 1543.78 | 0.00 | 0.17 |
| mad-cows | 5 | 1544.15 | 0.38 | 0.14 |
| brood size | 4 | 1544.17 | 0.40 | 0.14 |
| mad-cows + brood size | 6 | 1544.42 | 0.64 | 0.12 |
| order | 5 | 1545.66 | 1.88 | 0.07 |
| hatching date | 4 | 1545.74 | 1.96 | 0.06 |
| brood size + hatching date | 5 | 1546.08 | 2.30 | 0.05 |
| mad-cows + hatching date | 6 | 1546.17 | 2.39 | 0.05 |
| brood size + mad-cows + hatching date | 7 | 1546.40 | 2.63 | 0.04 |
| mad-cows + region | 8 | 1547.50 | 3.73 | 0.03 |
| order + hatching date | 6 | 1547.64 | 3.87 | 0.02 |
| mad-cows + order + hatching date | 8 | 1547.94 | 4.16 | 0.02 |
| brood size + mad-cows + region | 9 | 1548.20 | 4.42 | 0.02 |
| region | 6 | 1548.60 | 4.83 | 0.01 |
| brood size + region | 7 | 1549.31 | 5.53 | 0.01 |
| mad-cows + hatching date + region | 9 | 1549.54 | 5.76 | 0.01 |
| mad-cows + order + region | 10 | 1549.69 | 5.92 | 0.01 |
| mad-cows + brood size + hatching date + region | 10 | 1550.22 | 6.44 | 0.01 |
| hatching date | 7 | 1550.60 | 6.83 | 0.01 |
| order + region | 8 | 1550.80 | 7.02 | 0.01 |
| brood size + hatching date + region | 8 | 1551.26 | 7.48 | 0.00 |
| mad-cows + order + hatching date + region | 11 | 1551.73 | 7.96 | 0.00 |
| order + hatching date + region | 9 | 1552.81 | 9.03 | 0.00 |

Table S7. Alternative models (ΔAICc < 2; see Table 6) obtained to assess the effects of hatching date (hatching date), region (region) and food availability (mad-cows) on the probability of a nestling Egyptian Vulture being a male in peninsular Spain considering only first-hatched nestlings from double-broods (*n* = 324). All models were run including year and territory as random terms. In bold, significant effects (p<0.05).

| **Model** | **Variable** | **Estimate** | **SE** | **p-value** |
| --- | --- | --- | --- | --- |
| **mad-cows** | mad-cows (post) | 0.71 | 0.29 | **0.0142** |
|  | mad-cows (pre) | 0.37 | 0.30 | 0.2132 |
| **mad-cows + region** | mad-cows (post) | 0.67 | 0.32 | **0.0338** |
|  | mad-cows (pre) | 0.16 | 0.32 | 0.6070 |
|  | region (Aragon) | 0.38 | 0.41 | 0.3542 |
|  | region (Navarra) | 0.76 | 0.37 | **0.0371** |
|  | region (Segovia) | 0.31 | 0.41 | 0.4440 |
| **region** | region (Aragon) | 0.06 | 0.38 | 0.8695 |
|  | region (Navarra) | 0.70 | 0.35 | **0.0463** |
|  | region (Segovia) | 0.38 | 0.41 | 0.3598 |

Table S8. Models assessing the effects of food availability (mad-cows) and brood size (brood size) on the probability of a nestling Egyptian Vulture being a male in the Balearic Islands (*n* = 50). Estimates, standard errors (SE), and 95% confidence intervals (CI) were obtained after model averaging. All models were run including year and territory as random terms. The null model was included in our set of models. df: degrees of freedom; AICc: Akaike information criterion corrected for small sample sizes; ΔAICc: difference between the AICc of model *i* and that of the best model (i.e., the model with the lowest AICc); w: Akaike weight.

| Model selection | | | | |
| --- | --- | --- | --- | --- |
| Model | df | AICc | ΔAICc | w |
| brood size | 4 | 75.10 | 0.00 | 0.45 |
| null | 3 | 75.76 | 0.65 | 0.32 |
| brood size + mad-cows | 5 | 77.57 | 2.46 | 0.13 |
| mad-cows | 4 | 78.04 | 2.94 | 0.10 |
| Model averaging | | | | |
| Variable | Estimate | SE | 2.5% CI | 97.5% CI |
| Intercept | -0.10 | 0.35 | -0.79 | 0.60 |
| brood size (2) | 1.14 | 0.68 | -0.23 | 2.51 |

Table S9. Models assessing the effects of hatching date (hatching date), food availability (mad-cows), brood size (brood size), hatching order (order) and type of breeding unit (unit) on the probability of a nestling Egyptian Vulture being a male in the Canary Islands (*n* = 499). All models were run including year and territory as random terms. The null model was included in our set of models. df: degrees of freedom; AICc: Akaike information criterion corrected for small sample sizes; ΔAICc: difference between the AICc of model *i* and that of the best model (i.e., the model with the lowest AICc); w: Akaike weight.

| Model | df | AICc | ΔAICc | w |
| --- | --- | --- | --- | --- |
| brood size | 4 | 691.92 | 0.00 | 0.10 |
| order | 5 | 692.04 | 0.12 | 0.10 |
| null | 3 | 692.27 | 0.36 | 0.09 |
| unit | 5 | 692.76 | 0.85 | 0.07 |
| brood size + unit | 6 | 692.76 | 0.85 | 0.07 |
| order + unit | 7 | 692.88 | 0.97 | 0.06 |
| hatching date | 4 | 693.02 | 1.11 | 0.06 |
| brood size + hatching date | 5 | 693.08 | 1.16 | 0.06 |
| hatching date + unit | 6 | 693.21 | 1.30 | 0.05 |
| order + hatching date | 6 | 693.44 | 1.52 | 0.05 |
| brood size + hatching date + unit | 7 | 693.65 | 1.73 | 0.04 |
| order + hatching date + unit | 8 | 694.05 | 2.13 | 0.04 |
| brood size + mad-cows | 6 | 694.66 | 2.75 | 0.03 |
| order + mad-cows | 7 | 694.81 | 2.89 | 0.02 |
| mad-cows | 5 | 695.09 | 3.17 | 0.02 |
| brood size + mad-cows + unit | 8 | 695.48 | 3.56 | 0.02 |
| mad-cows + unit | 7 | 695.59 | 3.67 | 0.02 |
| order + mad-cows + unit | 9 | 695.62 | 3.70 | 0.02 |
| mad-cows + hatching date | 6 | 695.73 | 3.82 | 0.02 |
| brood size + mad-cows + hatching date | 7 | 695.76 | 3.84 | 0.02 |
| mad-cows + hatching date + unit | 8 | 695.87 | 3.96 | 0.01 |
| order + mad-cows + hatching date | 8 | 696.14 | 4.23 | 0.01 |
| brood size + mad-cows + hatching date + unit | 9 | 696.24 | 4.32 | 0.01 |
| order + mad-cows + hatching date + unit | 10 | 696.66 | 4.75 | 0.01 |

Table S10. Models assessing the effect of the annual conspecific density (density) on the annual offspring sex ratio of Egyptian Vultures from (a) the Canary (n = 499) and (b) the Balearic Islands (n = 50). The null model was included in our set of models. df: degrees of freedom; AICc: Akaike information criterion corrected for small sample sizes; ΔAICc: difference between the AICc of model *i* and that of the best model (i.e., the model with the lowest AICc); w: Akaike weight.

| Model | df | AICc | ΔAICc | w |
| --- | --- | --- | --- | --- |
| (a) Canary Islands |  |  |  |  |
| null | 2 | -29.96 | 0.00 | 0.79 |
| density | 3 | -27.33 | 2.63 | 0.21 |
| (b) Balearic Islands |  |  |  |  |
| null | 2 | 2.63 | 0.00 | 0.99 |
| density | 3 | 11.60 | 8.97 | 0.01 |

Table S11. Models assessing the effect of the annual population trend (as seven alternative variables, trends for intervals of one to seven years before; see Materials and Methods for details) on the annual offspring sex ratio of Egyptian Vultures from the Canary Islands (n = 499). The null model was included in our set of models. df: degrees of freedom; AICc: Akaike information criterion corrected for small sample sizes; ΔAICc: difference between the AICc of model *i* and that of the best model (i.e., the model with the lowest AICc); w: Akaike weight.

| Model | df | AICc | ΔAICc | w |
| --- | --- | --- | --- | --- |
| null | 2 | -21.22 | 0.00 | 0.36 |
| trend1 | 3 | -19.14 | 2.09 | 0.13 |
| trend2 | 3 | -18.63 | 2.59 | 0.10 |
| trend3 | 3 | -18.52 | 2.70 | 0.09 |
| trend6 | 3 | -18.36 | 2.87 | 0.08 |
| trend4 | 3 | -18.30 | 2.93 | 0.08 |
| trend7 | 3 | -18.28 | 2.94 | 0.08 |
| trend5 | 3 | -18.24 | 2.98 | 0.08 |

Table S12. Number of breeding females and pairs from the Canary Islands with each type of sex sequence in their known life as breeders. Each type of sex sequence refers to the number of consecutive individuals of the same sex reared by each individualised female or pair, including both single and double broods. If a female or pair was not seen during a certain year, we considered that particular sex sequence as interrupted. f: female; m: male.

| type of sex sequence | breeding females | breeding pairs |
| --- | --- | --- |
| 2f | 20 | 13 |
| 2m | 19 | 8 |
| 3f | 11 | 7 |
| 3m | 11 | 7 |
| 4f | 3 | 1 |
| 4m | 8 | 3 |
| 5f | 0 | 0 |
| 5m | 3 | 3 |

**2. Model fits**

We used the DHARMa package in R (Hartig, 2018) to evaluate the fit of the final models. DHARMa employed a simulation-based approach to create standardized residuals (values between 0 and 1) for fitted (generalized) linear (mixed) models and test the significance of the dispersion parameter, zero-inflation, and goodness-of-fit of the model (H_0_: fitted model fits the data well).


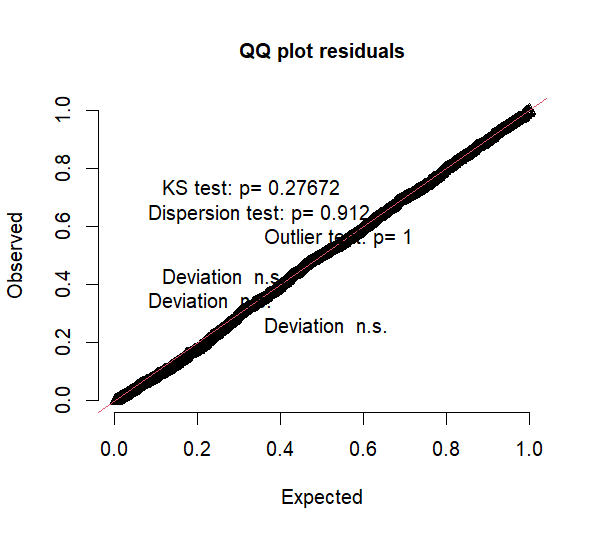

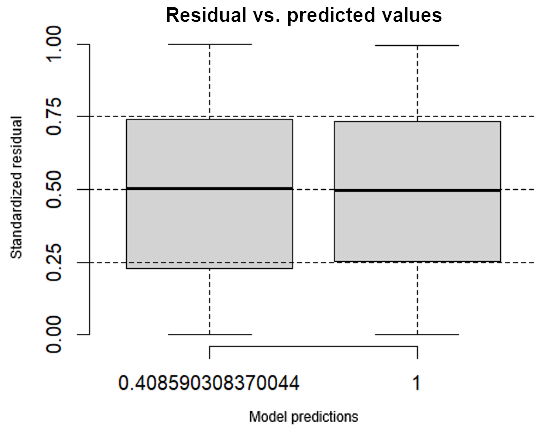


Figure S2. QQ-plot and standard residuals plot for the final model obtained to assess the effects of insularity on nestling sex, considering all nestlings (Table 4). No significant problems were detected.

**3. Other material**


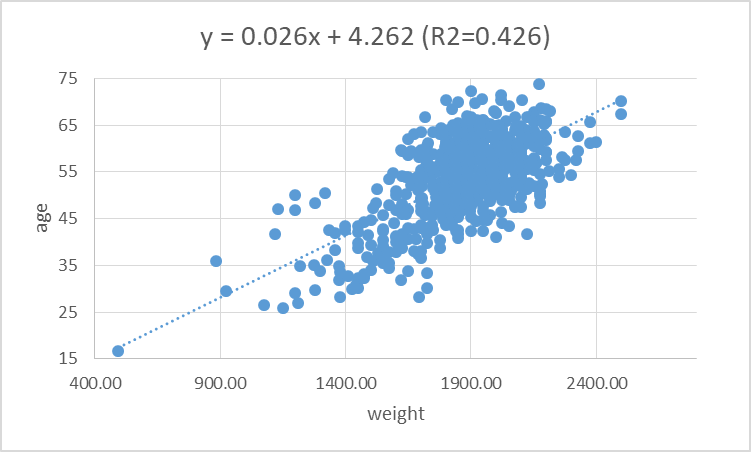


Figure S3. Linear regression of the weight of nestlings of known age from the broods in Navarra and Aragon used to estimate the age of the nestlings from Segovia, regardless of their hatching order (age = 0.026*weight + 4.262; R^2^ = 0.426).
